# Factors associated with balance impairments in the community-dwelling elderly in urban China

**DOI:** 10.1186/s12877-023-04219-z

**Published:** 2023-09-07

**Authors:** Qinghua Xia, Peng Zhou, Xia Li, Xiaofen Li, Lei Zhang, Xuefei Fan, Zhoulan Zhao, Yu Jiang, Jianhong Zhu, Hongmei Wu, Mengdi Zhang

**Affiliations:** 1 Changning Center for Disease Control and Prevention, Shanghai, 200051 China; 2 Longhua Hospital, Shanghai University of Traditional Chinese Medicine, Shanghai, 200032 China; 3 Institute of Nutrition and Diseases, Department of Preventive Medicine, Wenzhou Medical University, Wenzhou, 325035 China

**Keywords:** Elderly, Community-dwelling, Balance, Static balance, Postural stability, Dynamic balance, Factor

## Abstract

**Background:**

Identification of factors relevant to balance performance impairments in the elderly population was critical for developing effective interventions and preventions. However, there have been very limited data available based on large scale studies. The present study identified factors that independently contributed to performance impairments in overall balance, domains of static balance, postural stability, and dynamic balance, and individual items.

**Methods:**

A total of 1984 community-dwelling Chinese elderly from urban areas of Shanghai were recruited. Information on demographic characteristic, exercise, and health status were collected with a face-to-face interview. Balance performances were assessed on site by trained investigators based on the X16 balance testing scale. To identify the effectors, ordinal logistic regression analysis was applied for overall balance, static balance, postural stability, and dynamic balance. Binary logistic regression analysis was used for 16 items.

**Results:**

The community-dwelling elderly residents were aged from 60 to 97 years old. With increases of age, risks of impairments in overall balance increased gradually (ORs from 1.26 to 3.20, all *P* < 0.01). In the elderly with overweight and obesity, there was higher proportion of balance impairments compared to the elderly with normal BMI (OR = 1.26, *P* < 0.001). Regular exercise every week was associated with reduced risks of balance impairments (ORs from 0.63 to 0.73, all *P* < 0.001). Presences with vision lesion (ORs from 1.28 to 1.59, all *P* < 0.001), moderate hearing impairment (OR = 1.54, *P* < 0.001), somesthesis dysfunction (ORs from 1.59 to 13.26, all *P* < 0.001), and cerebrovascular disease (OR = 1.45, *P* = 0.001) were related to increased risks of balance impairments. Likewise, age, exercise, vision, hearing, somesthesis, and cerebrovascular disease were significantly associated with static balance, postural stability, and dynamic balance. Both overweight and obesity and underweight were associated with higher proportions of dynamic balance impairments. Regular exercise was significantly related to reduced risks of impairments in 15 out of the 16 items.

**Conclusions:**

In the elderly, age, overweight and obesity, exercise, vision, hearing, somesthesia, and cerebrovascular disease were dominant factors associated with impairments in overall balance, domains of static balance, postural stability, and dynamic balance, and most individual items.

**Trial registration:**

Not applicable.

**Supplementary Information:**

The online version contains supplementary material available at 10.1186/s12877-023-04219-z.

## Background

Population ageing has been a global phenomenon for decades. There was 702.9 million population aged 65 years and over in the world in 2019 and there will be 997.5 million in 2030. China had the largest population in the world, and accounted for about one fourth of the global elderly population [1]. In China, population aged 65 years and over was 11.9% of total population in 2018, the proportion was nearly doubled within 20 years [2]. Along with increasing life expectancy, accelerating ageing, and growing prevalence of chronic diseases in the elderly, their health status together with sequent socioeconomic issues have become significant public health challenges worldwide [3]. In the elderly, balance performance has been an important indicator of their functional status. Substantial evidence showed that functional declines in balance performance predicted poor health outcomes, reduced independence of activity of daily living, and elevated need for health care [4–7].

Maintenance of balance depended on responses and coordinations of the visual [8–11], vestibular [12, 13], somatosensory [14], muscle, and skeletal systems [15, 16]. With increasing age in the elderly, these physiological and functional conditions declined progressively, consequently, balance deficit manifested [16], falls and fall-related injuries occurred [9, 17, 18]. Besides, respiratory capacity, metabolic syndrome and related disorders [14, 19], cognition [20], functions of nervous system [13], and life styles [19] were involved in balance performance.

Identification of factors relevant to balance performance impairments in the elderly population was critical for developing effective interventions and preventions. The present study was based on community-dwelling elderly in urban of China. The sociodemographic, life style, heath characteristics, and disease conditions were investigated, their independent contributions to balance performances at overall balance, domain (static balance, postural stability, and dynamic balance), and individual item levels were assessed with multivariable regression analyses. The findings provided scientific evidence for guidance on solutions to balance impairments.

## Methods

### Subjects

A total of 1984 community-dwelling Chinese elderly from urban areas of Shanghai were recruited according to the previous report [21]. In brief, 2312 residents aged 60 years or over were investigated, out of which 328 elderly were not admitted due to the lacks of independencies in walking or cognitive status, or incompleteness of data collection. The remaining 1984 elderly were eligible and included for association analyses [21].

### Ethical consideration

The study was approved by Institutional Review Board (IRB) of Changning Center for Disease Control and Prevention, Shanghai. The protocols were in accordance with the guidelines of institutional declaration. The written informed consent forms were signed by the participants.

### Data collection

Data were collected with a face-to-face interview. Demographic information included gender, age, height, body weight, education level, exercise, and smoking. Body mass index (BMI) was calculated. Information on health status included vision, eye diseases (cataract, retinopathy), hearing, somesthesis, hypotension, hypertension, hyperlipidemia, cardiovascular disease, cerebrovascular disease, linguistic incompetence, varicose veins, diabetes, chronic bronchitis, asthma, arthritis, osteoporosis, intervertebral disc herniation, hemorrhoids, and prostate hypertrophy (Table 1).

**Table 1:** Demographic characteristics of the elderly and value assignments for variables

Type of variables	Variables	Categories	Value assigned	n	%
X	Gender	Male	1 (ref)	940	47.4
		Female	2	1044	52.6
	Age (yrs)	60-	6 (ref)	511	25.8
		65-	5	530	26.7
		70-	4	378	19.1
		75-	3	297	15.0
		80-	2	165	8.3
		85–97	1	103	5.2
	Education	Elementary school or below	1	204	10.3
		Middle or high school	2	1022	51.5
		College or above	3 (ref)	758	38.2
	Income^a^	0-	1 (ref)	12	0.6
		500-	2	166	8.4
		2000-	3	716	36.1
		3000-	4	404	20.4
		4000-	5	275	13.9
		5000-	6	276	13.9
		10,000-	7	135	6.8
	BMI (kg/m^2^)^b^	Underweight (< 18.5)	1	63	3.2
		Overweight or obesity [24.0-)	2	746	37.6
		Normal [18.5–24.0)	3 (ref)	1174	59.2
	Exercise	0-	4 (ref)	453	22.8
	(times/week, > 10 min/time)	1-	3	442	22.3
		4-	2	244	12.3
		7	1	845	42.6
	Smoking	No	0 (ref)	1575	79.4
		Yes	1	409	20.6
	Vision (m)^c^	4-	3 (ref)	1348	67.9
		1-	2	483	24.3
		0-	1	153	7.7
	Eye diseases	No	0 (ref)	1811	91.3
	(Cataract or retinopathy)	Yes	1	173	8.7
	Hearing impairment^d^	0	4 (ref)	1589	80.1
		Moderate	3	332	16.7
		Severe	2	54	2.7
		Profound	1	9	0.5
	Somatosensory dysfunction	0	4 (ref)	1405	70.8
		Mild	3	476	24.0
		Moderate	2	93	4.7
		Severe	1	10	0.5
	Hypotension	No	0 (ref)	1961	98.8
		Yes	1	23	1.2
	Hypertension	No	0 (ref)	1047	52.8
		Yes	1	937	47.2
	Hyperlipidemia	No	0 (ref)	1735	87.4
		Yes	1	249	12.6
	Cardiovascular disease	No	0 (ref)	1706	86.0
		Yes	1	278	14.0
	Cerebrovascular disease	No	0 (ref)	1852	93.3
		Yes	1	132	6.7
	Linguistic incompetence	No	0 (ref)	1930	97.3
		Yes	1	54	2.7
	Varicose veins	No	0 (ref)	1931	97.3
		Yes	1	53	2.7
	Diabetes	No	0 (ref)	1736	87.5
		Yes	1	248	12.5
	Chronic bronchitis	No	0 (ref)	1893	95.4
		Yes	1	91	4.6
	Asthma	No	0 (ref)	1958	98.7
		Yes	1	26	1.3
	Arthritis	No	0 (ref)	1779	89.7
		Yes	1	205	10.3
	Osteoporosis	No	0 (ref)	1794	90.4
		Yes	1	190	9.6
	Intervertebral disc herniation	No	0 (ref)	1880	94.8
		Yes	1	104	5.2
	Hemorrhoids	No	0 (ref)	1863	93.9
		Yes	1	121	6.1
	Prostate hypertrophy	No	0 (ref)	1895	95.5
		Yes	1	89	4.5
Y (score)^e^	Balance performance^f^	Good (18–20)	1 (ref)	1484	74.8
		Fair (11–17)	2	355	17.9
		Poor (0–10)	3	145	7.3
	Static balance	4	1 (ref)	1462	73.7
		3	2	270	13.6
		2	3	101	5.1
		1	4	42	2.1
		0	5	109	5.5
	Postural stability	8	1 (ref)	1413	71.2
		7	2	92	4.6
		6	3	171	8.6
		5	4	73	3.7
		4	5	180	9.1
		3	6	17	0.9
		2	7	18	0.9
		1	8	4	0.2
		0	9	16	0.8
	Dynamic balance	8	1 (ref)	1505	75.9
		7	2	206	10.4
		6	3	68	3.4
		5	4	31	1.6
		4	5	30	1.5
		3	6	27	1.4
		2	7	21	1.1
		1	8	25	1.3
		0	9	71	3.6
	I 1	1	0 (ref)	1826	92.0
		0	1	158	8.0
	I 2	1	0 (ref)	1815	91.5
		0	1	169	8.5
	I 3	1	0 (ref)	1758	88.6
		0	1	226	11.4
	I 4	1	0 (ref)	1503	75.8
		0	1	481	24.2
	II 5	2	0 (ref)	1727	87.0
		1 or 0	1	257	13.0
	II 6	2	0 (ref)	1681	84.7
		1 or 0	1	303	15.3
	II 7	2	0 (ref)	1510	76.1
		1 or 0	1	474	23.9
	II 8	2	0 (ref)	1444	72.8
		1 or 0	1	540	27.2
	III 9	1	0 (ref)	1817	91.6
		0	1	167	8.4
	III 10	1	0 (ref)	1769	89.2
		0	1	215	10.8
	III 11	1	0 (ref)	1665	83.9
		0	1	319	16.1
	III 12	1	0 (ref)	1794	90.4
		0	1	190	9.6
	III 13	1	0 (ref)	1772	89.3
		0	1	212	10.7
	III 14	1	0 (ref)	1865	94.0
		0	1	119	6.0
	III 15	1	0 (ref)	1844	92.9
		0	1	140	7.1
	III 16	1	0 (ref)	1787	90.1
		0	1	197	9.9

^a^Income was the average Chinese yuan per month *per capita*. ^b^BMI (kg/m2) was categorized into underweight, normal, and overweight and obesity. ^c^Vision, could be with the aid of glasses, vision was classified into 3 categories, without difficulty for further than 4 m (4-), without difficulty for 1–4 m (1-), able for less than 1 m (0-). ^d^Hearing, could be with the aid of audiphone. ^e^Y were categorized based on the score (points) evaluated by the X16 scale. ^f^The overall balance performance was categorized into 3 groups with two-step cluster analysis

Balance performances were assessed on site by trained investigators based on the X16 balance testing scale (the X16 scale). Domains of static balance, postural stability, and dynamic balance, were manifested with 4, 4, and 8 items, respectively. The performance on each item was evaluated, then performances on domains and overall balance were obtained [22].

### Value assignments for variables

Value assignments for independent (X) and dependent (Y) variables were listed in Table 1. Ages were divided into a total of 6 groups. Ages from 60 to 85 years old made 5 groups by a 5-year interval, the 6th group was composed of all the elderly ages 85 years old and over given relatively limited sample size.

The BMI (kg/m^2^) was categorized into 3 groups of underweight, normal, and overweight and obesity. The cutoff values were according to guidelines for prevention and control of overweight and obesity in Chinese adults [23].

Vision could be with the aid of glasses. Vision was classified into 3 categories, without difficulty for further than 4 m (4-) was valued as 3, without difficulty for 1–4 m (1-) was valued as 2, and able for less than 1 m (0-) was valued as 1.

Hearing could be with the aid of audiphone. Hearing was classified based on WHO’s grades of hearing impairments with modifications [24]. 0, no impairment or slight hearing problems, able to hear words in normal voice, was valued as 4. Moderate impairment, able to hear words using raised voice, was valued as 3. Severe impairment, able to hear words when shouted into better ear, was valued as 2. Profound impairment, unable to hear even a shouted voice, was valued as 1.

Without a certain disease (including disorder, dysfunction, or impairment) was valued as 0 while with a certain disease was assigned value of 1. Diseases with prevalence less than 1% were ruled out in analysis.

Dependent variables Y were categorized based on the score (points) evaluated by the X16 scale [22]. The overall balance performance was categorized into 3 groups (Good, fair, and poor) with two-step cluster analysis [21]. For overall balance performance and individual domains, good or intact balance performance was assigned value of 1, increased values were assigned with increasing impairments (correspondingly decreasing balance scores). For individual items, intact balance was assigned value of 0, and lesioned balance was valued 1.

Independent variables of men, younger age, higher education level, normal function, and without disease were set as references. Dependent variables of good or intact balance performance condition were set as references.

### Statistical analyses

EpiData 3.0 (The EpiData Association, Odense, Denmark) and SPSS 23.0 (SPSS Inc. Chicago, IL, USA) were used for data entry and data analysis, respectively.

Ordinal logistic regression analysis was adopted to identify the effectors of overall balance performance and individual domains (static balance, postural stability, and dynamic balance). Binary logistic regression analysis was used for 16 items.

For each dependent variable, univariate logistic regression mode was applied to list out potentially associated independent variables with significance level of 0.10 (Tables S1-S7), then stepwise multivariable logistic regression analysis was performed to identify associations with significance level of 0.05.

The regression coefficient (*β*), odds ratios (OR), and the 95% confidence intervals (CI) were reported.

## Results

### Demographic characteristics of the elderly

A total of 1984 community-dwelling elderly were included in the study, their demographic characteristics were summarized in Table 1. The elderly residents were aged from 60 to 97 years old, amongst 1041 (52.5%) residents were aged from 60 to 70 years, 675 (34.1%) were within 70 to 80 years, and 268 (13.5%) were aged over 80 years. There were 940 (47.4%) men and 1044 (52.6%) women.

Of the elderly, 1174 (59.2%) residents maintained normal BMI, 746 (37.6%) residents were overweight or obese, and 63 (3.2%) residents were underweight. Among the elderly, 636 (32.0%) residents had vision lesion, 173 (8.7%) residents had eye diseases, 395 (19.9%) residents had hearing impairments, and 579 (29.2%) residents had somesthesis disorders.

### Factors associated with impairments in overall balance performance

Age, BMI, exercise, vision, hearing, somesthesis, and cerebrovascular disease were associated with impairments in overall balance (Table 2). Compared to the elderly aged 60- years, risks of balance impairments for increasing aged elderly elevated gradually, ORs increased from 1.26 for the elderly aged 65- years to 3.20 for those aged 85 years and above (All *P* < 0.01). Compared to the elderly with normal BMI, there was higher proportion of balance impairments in the elderly with overweight and obesity (OR = 1.26, *P* < 0.001), whereas there was no significant alteration in the elderly with underweight.

**Table 2:** Factors associated with balance impairments

Variable	β	SE	Waldc^2^	*P*	OR	95% CI
Age (yrs)						
60-	ref					
65-	0.228	0.082	7.701	0.006	1.26	(1.07, 1.48)
70-	0.313	0.088	12.635	< 0.001	1.37	(1.15, 1.63)
75-	0.530	0.094	31.977	< 0.001	1.70	(1.41, 2.04)
80-	0.768	0.116	44.035	< 0.001	2.15	(1.72, 2.70)
85–97	1.164	0.150	59.911	< 0.001	3.20	(2.39, 4.30)
Income	-0.074	0.020	13.665	< 0.001	0.93	(0.89, 0.97)
BMI (kg/m^2^)						
Overweight or obesity [24.0-)	0.230	0.059	15.006	< 0.001	1.26	(1.12, 1.41)
Normal [18.5–24.0)	ref					
Exercise (times/week, > 10 min/time)						
0-	ref					
1-	-0.317	0.084	14.412	< 0.001	0.73	(0.62, 0.86)
4-	-0.469	0.102	21.064	< 0.001	0.63	(0.51, 0.76)
7-	-0.405	0.074	29.547	< 0.001	0.67	(0.58, 0.77)
Vision (m)						
4-	ref					
1-	0.248	0.069	12.939	< 0.001	1.28	(1.12, 1.47)
0-	0.466	0.110	17.869	< 0.001	1.59	(1.28, 1.98)
Hearing impairment						
0	ref					
Moderate	0.429	0.081	27.821	< 0.001	1.54	(1.31, 1.80)
Somatosensory dysfunction						
0	ref					
Mild	0.461	0.070	43.037	< 0.001	1.59	(1.38, 1.82)
Moderate	1.580	0.178	79.158	< 0.001	4.85	(3.43, 6.88)
Severe	2.585	0.733	12.443	< 0.001	13.26	(3.15, 55.74)
Cerebrovascular disease	0.372	0.114	10.579	0.001	1.45	(1.16, 1.81)

Non-significant factors were not presented. Significance level is 0.05

Presences with vision lesion (all *P* < 0.001), moderate hearing impairment (OR = 1.54, *P* < 0.001), and cerebrovascular disease (OR = 1.45, *P* = 0.001) were related to increased risks of balance impairments.

Aggravating somatosensory dysfunctions were related to distinct increase in risks of balance impairments, with ORs of 1.59, 4.85, and 13.26 for mild, moderate, and severe dysfunctions, respectively (all *P* < 0.001).

Relative to low income, income increase was negatively associated with risks of balance impairments (OR = 0.93, *P* < 0.001).

Relative to no exercise, regular exercises of 1 to 7 times every week were associated with reduced risks of balance impairments, the ORs were from 0.63 to 0.73 (All *P* < 0.001).

### Factors associated with impairments in static balance, postural stability, and dynamic balance

Similar to associations with impairments in overall balance, age, exercise, vision, hearing, somesthesis, and cerebrovascular disease were associated with impairments in all 3 domains of static balance (Table 3), postural stability (Table 4), and dynamic balance (Table 5). The positive and negative associations were indicated (Table 6).

**Table 3:** Factors associated with static balance impairments

Variable	β	SE	Waldc^2^	*P*	OR	95% CI
Age (yrs)						
60-	ref					
70-	0.249	0.081	9.569	0.002	1.28	(1.10, 1.50)
75-	0.400	0.086	21.458	< 0.001	1.49	(1.26, 1.77)
80-	0.448	0.109	17.031	< 0.001	1.56	(1.27, 1.94)
85–97	0.628	0.140	20.117	< 0.001	1.87	(1.42, 2.47)
Education						
Elementary school or below	0.302	0.099	9.377	0.002	1.35	(1.11, 1.64)
College or above	ref					
Income	-0.046	0.019	5.921	0.015	0.96	(0.92, 0.99)
BMI (kg/m^2^)						
Underweight (< 18.5)	0.253	0.148	2.902	0.088	1.29	(0.96, 1.72)
Normal [18.5–24.0)	ref					
Exercise (times/week, > 10 min/time)						
0-	ref					
1-	-0.334	0.078	18.551	< 0.001	0.72	(0.61, 0.83)
4-	-0.526	0.095	30.677	< 0.001	0.59	(0.49, 0.71)
7-	-0.332	0.068	23.552	< 0.001	0.72	(0.63, 0.82)
Vision (m)						
4-	ref					
1-	0.150	0.064	5.538	0.019	1.16	(1.03, 1.32)
0-	0.381	0.103	13.817	< 0.001	1.46	(1.20, 1.79)
Hearing impairment						
0	ref					
Moderate	0.232	0.076	9.219	0.002	1.26	(1.09, 1.46)
Severe	0.364	0.184	3.914	0.048	1.44	(1.00, 2.06)
Somatosensory dysfunction						
0	ref					
Mild	0.152	0.067	5.196	0.023	1.16	(1.02, 1.33)
Moderate	0.683	0.143	22.634	< 0.001	1.98	(1.49, 2.62)
Severe	1.449	0.528	7.522	0.006	4.26	(1.51, 11.99)
Hypertension	0.121	0.053	5.119	0.024	1.13	(1.02, 1.25)
Hyperlipidemia	0.137	0.080	2.910	0.088	1.15	(0.98, 1.34)
Cerebrovascular disease	0.179	0.107	2.793	0.095	1.20	(0.97, 1.48)

Non-significant factors were not presented. Significance level is 0.05

**Table 4:** Factors associated with postural stability impairments

Variable	β	SE	Waldc^2^	*P*	OR	95% CI
Age (yrs)						
60-	ref					
65-	0.249	0.074	11.303	0.001	1.28	(1.11, 1.48)
70-	0.375	0.081	21.643	< 0.001	1.46	(1.24, 1.70)
75-	0.589	0.086	46.872	< 0.001	1.80	(1.52, 2.13)
80-	0.664	0.106	39.074	< 0.001	1.94	(1.58, 2.39)
85–97	1.103	0.135	66.898	< 0.001	3.01	(2.31, 3.93)
Income	-0.040	0.018	4.598	0.032	0.96	(0.93, 1.00)
BMI (kg/m^2^)						
Overweight or obesity [24.0-)	0.217	0.053	16.631	< 0.001	1.24	(1.12, 1.38)
Normal [18.5–24.0)	ref					
Exercise (times/week, > 10 min/time)						
0-	ref					
1-	-0.228	0.075	9.146	0.002	0.80	(0.69, 0.92)
4-	-0.286	0.091	9.884	0.002	0.75	(0.63, 0.90)
7-	-0.353	0.068	27.197	< 0.001	0.70	(0.61, 0.80)
Vision (m)						
4-	ref					
1-	0.134	0.063	4.568	0.033	1.14	(1.01, 1.29)
0-	0.216	0.099	4.788	0.029	1.24	(1.02, 1.51)
Hearing impairment						
0	ref					
Moderate	0.263	0.074	12.664	< 0.001	1.30	(1.13, 1.50)
Somatosensory dysfunction						
0	ref					
Mild	0.521	0.065	63.524	< 0.001	1.68	(1.48, 1.91)
Moderate	1.161	0.135	73.830	< 0.001	3.19	(2.45, 4.16)
Severe	2.150	0.430	25.043	< 0.001	8.58	(3.7, 19.91)
Cerebrovascular disease	0.448	0.101	19.497	< 0.001	1.57	(1.28, 1.91)
Linguistic incompetence	0.352	0.163	4.664	0.031	1.42	(1.03, 1.96)

Non-significant factors were not presented. Significance level is 0.05

**Table 5:** Factors associated with dynamic balance impairments

Variable	β	SE	Waldc^2^	*P*	OR	95% CI
Age (yrs)						
60-	ref					
65-	0.155	0.074	4.336	0.037	1.17	(1.01, 1.35)
70-	0.182	0.081	5.053	0.025	1.20	(1.02, 1.41)
75-	0.317	0.086	13.598	< 0.001	1.37	(1.16, 1.62)
80-	0.526	0.105	25.046	< 0.001	1.69	(1.38, 2.08)
85–97	0.948	0.134	50.220	< 0.001	2.58	(1.99, 3.35)
Income	-0.080	0.019	18.531	< 0.001	0.92	(0.89, 0.96)
BMI (kg/m^2^)						
Underweight (< 18.5)	0.270	0.147	3.384	0.066	1.31	(0.98, 1.75)
Overweight or obesity [24.0-)	0.217	0.054	15.844	< 0.001	1.24	(1.12, 1.38)
Normal [18.5–24.0)	ref					
Exercise (times/week, > 10 min/time)						
0-	ref					
4-	-0.208	0.092	5.161	0.023	0.81	(0.68, 0.97)
7-	-0.358	0.069	26.932	< 0.001	0.70	(0.61, 0.80)
Vision (m)						
4-	ref					
1-	0.235	0.063	13.770	< 0.001	1.27	(1.12, 1.43)
0-	0.331	0.100	10.951	0.001	1.39	(1.14, 1.69)
Hearing impairment						
0	ref					
Moderate	0.376	0.075	25.378	< 0.001	1.46	(1.26, 1.69)
Somatosensory dysfunction						
0	ref					
Mild	0.330	0.065	25.678	< 0.001	1.39	(1.22, 1.58)
Moderate	1.175	0.146	64.995	< 0.001	3.24	(2.43, 4.31)
Severe	1.805	0.491	13.544	< 0.001	6.08	(2.33, 15.91)
Cerebrovascular disease	0.259	0.104	6.170	0.013	1.30	(1.06, 1.59)
Linguistic incompetence	0.413	0.175	5.561	0.018	1.51	(1.07, 2.13)

Non-significant factors were not presented. Significance level is 0.05

**Table 6:** Factors associated with impairments in static balance, postural stability, dynamic balance, and balance

Variable	Static balance	Postural stability	Dynamic balance	Balance
Gender				
Age (yrs)				
60-				
65-		**+**	**+**	**+**
70-	**+**	**+**	**+**	**+**
75-	**+**	**+**	**+**	**+**
80-	**+**	**+**	**+**	**+**
85–97	**+**	**+**	**+**	**+**
Education				
Elementary school or below	**+**			
Middle or high school				
College or above				
Income	**-**	**-**	**-**	**-**
BMI (kg/m^2^)				
Underweight (< 18.5)				
Overweight or obesity [24.0-)		**+**	**+**	**+**
Normal [18.5–24.0)				
Exercise (times/week, > 10 min/time)				
0-				
1-	**-**	**-**		**-**
4-	**-**	**-**	**-**	**-**
7-	**-**	**-**	**-**	**-**
Smoking				
Vision (m)				
4-				
1-	**+**	**+**	**+**	**+**
0-	**+**	**+**	**+**	**+**
Eye diseases				
Hearing impairment				
0				
Moderate	**+**	**+**	**+**	**+**
Severe	**+**			
Profound				
Somatosensory dysfunction				
0				
Mild	**+**	**+**	**+**	**+**
Moderate	**+**	**+**	**+**	**+**
Severe	**+**	**+**	**+**	**+**
Hypotension				
Hypertension	**+**			
Hyperlipidemia				
Cardiovascular disease				
Cerebrovascular disease		**+**	**+**	**+**
Linguistic incompetence		**+**	**+**	
Varicose veins				
Diabetes				
Chronic bronchitis				
Asthma				
Arthritis				
Osteoporosis				
Intervertebral disc herniation				
Hemorrhoids				
Prostate hypertrophy				

+ OR > 1, positively associated with balance impairments, potential risk factors for balance impairments. - OR < 1, negatively associated with balance impairments, potential protective factors for balance impairments. Non-significant factors were not presented. Significance level is 0.05

Compared to the elderly aged 60- years, risks of impairments in static balance increased significantly since 70- years (OR from 1.28 to 1.87, all *P* < 0.005).

With increases of age, risks of impairments in postural stability (OR from 1.28 to 3.01, all *P* < 0.005) and dynamic balance (OR from 1.17 to 2.58, all *P* < 0.05) increased gradually and stably.

Compared to the elderly with normal BMI, there was higher proportions of postural stability and dynamic balance impairments in the elderly with overweight and obesity (both ORs = 1.24, both *P* < 0.001).

Distinct vision lesion (only able for less than 1 m, 0-) increased risks of impairments in static balance (OR = 1.46, *P* < 0.001), postural stability (OR = 1.24, *P* = 0.029), and dynamic balance (OR = 1.39, *P* = 0.001). Moderate hearing loss increased risks of impairments in static balance (OR = 1.26, *P* = 0.002), postural stability (OR = 1.30, *P* < 0.001), and dynamic balance (OR = 1.46, *P* < 0.001).

Aggravating somatosensory dysfunction was positively associated with distinct increase in risks of impairments in static balance (with ORs of 1.16, 1.98, and 4.26 for mild, moderate, and severe dysfunctions, respectively, all *P* < 0.05), postural stability (with ORs of 1.68, 3.19, and 8.58, all *P* < 0.001), and dynamic balance (with ORs of 1.39, 3.24, and 6.08, all *P* < 0.001).

Cerebrovascular disease was positively associated with impairments in static balance (OR = 1.20, *P* = 0.095), postural stability (OR = 1.57, *P* < 0.001), and dynamic balance (OR = 1.30, *P* = 0.013).

Compared to the elderly with low income, those with higher income had lower risks of impairments in static balance (OR = 0.96, *P* = 0.015), postural stability (OR = 0.96, *P* = 0.032), and dynamic balance (OR = 0.92, *P* < 0.001). Likewise, compared to the elderly without exercise, those with regular exercise of 1 to 7 times every week had lower risks of impairments in static balance (ORs from 0.59 to 0.72, all *P* < 0.001) and postural stability (ORs from 0.70 to 0.80, all *P* < 0.005), and exercise of 4 to 7 times every week had lower risks of impairments in dynamic balance (ORs from 0.70 to 0.81, both *P* < 0.05).

### Factors associated with impairments in individual items of balance

Further, potential factors associated with individual items of static balance (Table 7), postural stability (Table 8), and dynamic balance (Table 9) were identified. The positive and negative associations were indicated (Table 10).

**Table 7:** Factors associated with impairments in individual items of static balance

Variable	I 1	I 2	I 3	I 4
Age (yrs)				
60-		ref	ref	ref
65-				1.45 (0.99, 2.12)
70-			1.75 (1.00, 3.07)	2.31 (1.57, 3.40)
75-		2.16 (1.17, 3.99)	2.44 (1.41, 4.24)	3.58 (2.42, 5.30)
80-		2.72 (1.39, 5.29)	3.07 (1.67, 5.65)	3.58 (2.25, 5.70)
85–97		3.10 (1.48, 6.50)	4.22 (2.13, 8.36)	6.4 (3.67, 11.16)
Education				
Elementary school or below	2.06 (1.26, 3.38)		1.73 (1.06, 2.83)	1.86 (1.24, 2.79)
Middle or high school				1.42 (1.09, 1.86)
College or above	ref		ref	ref
Income		0.85 (0.74, 0.96)	0.85 (0.76, 0.96)	0.93 (0.85, 1.01)
BMI (kg/m^2^)				
Underweight (< 18.5)		2.11 (0.96, 4.63)	2.38 (1.18, 4.79)	1.68 (0.91, 3.10)
Overweight or obesity [24.0-)		1.37 (0.95, 1.97)	1.32 (0.95, 1.83)	
Normal [18.5–24.0)		ref	ref	ref
Exercise (times/week, > 10 min/time)				
0-	ref	ref	ref	ref
1-	0.54 (0.33, 0.88)	0.39 (0.24, 0.64)		0.44 (0.32, 0.61)
4-	0.39 (0.19, 0.82)	0.29 (0.13, 0.62)	0.36 (0.18, 0.69)	0.27 (0.17, 0.42)
7-	0.59 (0.38, 0.90)	0.48 (0.31, 0.73)	0.52 (0.35, 0.78)	0.40 (0.30, 0.53)
Vision (m)				
4-		ref	ref	ref
1-				1.53 (1.17, 2.01)
0-		1.79 (1.05, 3.05)	2.36 (1.46, 3.81)	1.79 (1.18, 2.70)
Hearing impairment				
0	ref	ref	ref	
Moderate	1.78 (1.15, 2.76)	1.64 (1.05, 2.55)	2.11 (1.45, 3.09)	
Severe	2.68 (1.30, 5.53)			
Somatosensory dysfunction				
0	ref	ref	ref	ref
Mild			1.40 (0.96, 2.04)	1.81 (1.39, 2.36)
Moderate	5.71 (3.27, 9.95)	5.24 (2.95, 9.32)	3.15 (1.79, 5.55)	3.32 (1.98, 5.57)
Severe	12.83 (2.91, 56.53)	21.53 (3.89, 119.05)	10.05 (2.05, 49.27)	5.29 (1.05, 26.61)
Hypertension	1.69 (1.18, 2.43)	1.43 (1.00, 2.06)		1.26 (1.00, 1.60)
Linguistic incompetence			1.90 (0.93, 3.86)	
Chronic bronchitis				1.66 (1.01, 2.72)
Asthma	2.79 (1.06, 7.38)			

Non-significant factors were not presented. Significance level is 0.05

**Table 8:** Factors associated with impairments in individual items of postural stability

Variable	II 5	II 6	II 7	II 8
Age (yrs)				
60-	ref	ref	ref	ref
65-			2.09 (1.39, 3.14)	1.72 (1.20, 2.48)
70-			2.56 (1.68, 3.90)	2.21 (1.52, 3.22)
75-	3.88 (2.16, 6.97)	2.92 (1.74, 4.92)	4.31 (2.82, 6.60)	3.29 (2.23, 4.85)
80-	6.06 (3.22, 11.38)	3.30 (1.83, 5.95)	5.95 (3.66, 9.67)	3.68 (2.32, 5.84)
85–97	9.35 (4.65, 18.77)	7.90 (4.05, 15.41)	7.62 (4.25, 13.65)	5.85 (3.34, 10.27)
Income	0.87 (0.77, 0.97)	0.91 (0.82, 1.02)	0.92 (0.85, 1.01)	
BMI (kg/m^2^)				
Underweight (< 18.5)	2.16 (1.01, 4.63)	1.86 (0.90, 3.84)		
Overweight or obesity [24.0-)	1.93 (1.38, 2.72)	1.87 (1.36, 2.56)	1.64 (1.27, 2.11)	1.82 (1.43, 2.32)
Normal [18.5–24.0)	ref	ref	ref	ref
Exercise (times/week, > 10 min/time)				
0-	ref		ref	ref
1-	0.67 (0.44, 1.03)			0.70 (0.50, 0.97)
4-	0.51 (0.28, 0.94)		0.66 (0.43, 1.02)	0.56 (0.37, 0.85)
7-	0.44 (0.29, 0.67)		0.57 (0.41, 0.78)	0.55 (0.41, 0.74)
Vision (m)				
4-	ref	ref	ref	ref
1-	1.67 (1.15, 2.43)	1.52 (1.07, 2.14)		1.61 (1.23, 2.11)
0-	2.00 (1.18, 3.38)		1.69 (1.09, 2.61)	1.84 (1.20, 2.80)
Hearing impairment				
0	ref	ref	ref	ref
Moderate	2.04 (1.39, 2.99)	2.41 (1.68, 3.44)	1.49 (1.09, 2.03)	1.63 (1.20, 2.20)
Somatosensory dysfunction				
0	ref	ref	ref	ref
Mild	2.31 (1.59, 3.37)	3.55 (2.52, 5.02)	2.86 (2.17, 3.77)	2.43 (1.86, 3.17)
Moderate	13.10 (7.19, 23.85)	16.01 (8.90, 28.82)	12.40 (6.65, 23.13)	9.59 (5.13, 17.92)
Severe	83.34 (8.85, 784.95)	170.76 (13.92, 2094.03)	34.49 (3.60, 330.24)	
Hypotension				4.03 (1.51, 10.75)
Cerebrovascular disease	1.78 (1.06, 3.00)	2.37 (1.46, 3.83)	1.71 (1.10, 2.66)	1.71 (1.11, 2.63)
Chronic bronchitis		1.69 (0.93, 3.06)		

Non-significant factors were not presented. Significance level is 0.05

**Table 9:** Factors associated with impairments in individual items of dynamic balance

Variable	III 9	III 10	III 11	III 12
Age (yrs)				
60-	ref	ref	ref	ref
65-		1.82 (0.97, 3.42)		
75-	2.93 (1.44, 5.96)	2.55 (1.35, 4.82)	1.79 (1.12, 2.86)	2.28 (1.19, 4.38)
80-	3.53 (1.63, 7.64)	2.77 (1.38, 5.56)	2.81 (1.66, 4.73)	3.32 (1.65, 6.68)
85–97	5.52 (2.45, 12.43)	5.89 (2.82, 12.29)	4.21 (2.30, 7.71)	5.26 (2.49, 11.10)
Education				
Elementary school or below			1.61 (1.02, 2.54)	
College or above			ref	
Income		0.88 (0.78, 0.99)	0.91 (0.82, 1.01)	0.84 (0.74, 0.96)
BMI (kg/m^2^)				
Underweight (< 18.5)	2.26 (0.94, 5.45)			
Overweight or obesity [24.0-)	2.96 (1.96, 4.48)	2.07 (1.44, 2.96)	1.35 (1.02, 1.79)	1.55 (1.07, 2.24)
Normal [18.5–24.0)	ref	ref	ref	ref
Exercise (times/week, > 10 min/time)				
0-	ref	ref	ref	ref
1-				0.58 (0.37, 0.93)
4-			0.53 (0.32, 0.89)	0.49 (0.25, 0.96)
7-	0.37 (0.22, 0.62)	0.29 (0.18, 0.47)	0.53 (0.37, 0.75)	0.43 (0.27, 0.68)
Vision (m)				
4-	ref	ref	ref	ref
1-	2.40 (1.54, 3.74)	1.80 (1.22, 2.67)	1.95 (1.43, 2.66)	2.14 (1.43, 3.19)
0-	2.79 (1.51, 5.17)	2.56 (1.50, 4.39)	2.32 (1.48, 3.63)	
Eye diseases	1.66 (0.94, 2.92)			
Hearing impairment				
0	ref	ref	ref	ref
Moderate	1.88 (1.20, 2.96)	2.59 (1.74, 3.85)	1.73 (1.24, 2.43)	2.84 (1.88, 4.28)
Somatosensory dysfunction				
0	ref	ref	ref	ref
Mild	1.55 (0.97, 2.47)	2.28 (1.52, 3.42)	1.36 (0.98, 1.88)	1.62 (1.05, 2.48)
Moderate	10.66 (5.80, 19.58)	8.88 (4.92, 16.00)	5.04 (2.95, 8.62)	7.96 (4.44, 14.29)
Severe	37.61 (5.81, 243.29)	36.62 (6.08, 220.54)	24.82 (2.73, 225.88)	31.43 (5.50, 179.68)
Hypertension		1.56 (1.09, 2.24)		
Cerebrovascular disease		1.89 (1.11, 3.20)		
Linguistic incompetence	2.60 (1.18, 5.73)		1.85 (0.92, 3.70)	2.13 (1.02, 4.45)

Non-significant factors were not presented. Significance level is 0.05

**Table 9:** Factors associated with impairments in individual items of dynamic balance (Continued)

Variable	III 13	III 14	III 15	III 16
Age (yrs)				
60-	ref	ref	ref	ref
70-				1.90 (0.95, 3.79)
75-	4.08 (2.16, 7.71)	2.13 (0.92, 4.92)	2.07 (0.92, 4.66)	2.72 (1.38, 5.35)
80-	5.19 (2.60, 10.37)	2.53 (1.03, 6.19)	3.63 (1.56, 8.44)	3.16 (1.51, 6.61)
85–97	8.57 (4.13, 17.81)	4.69 (1.84, 11.94)	4.66 (1.92, 11.31)	5.92 (2.72, 12.92)
Income	0.81 (0.71, 0.92)			
BMI (kg/m^2^)				
Overweight or obesity [24.0-)	1.90 (1.33, 2.72)		1.94 (1.25, 3.00)	1.77 (1.20, 2.61)
Normal [18.5–24.0)	ref		ref	ref
Exercise (times/week, > 10 min/time)				
0-	ref	ref	ref	ref
1-	0.60 (0.38, 0.93)		0.49 (0.29, 0.82)	0.42 (0.27, 0.67)
4-	0.42 (0.22, 0.82)		0.30 (0.13, 0.72)	0.45 (0.24, 0.86)
7-	0.36 (0.23, 0.56)	0.29 (0.15, 0.56)	0.21 (0.12, 0.39)	0.19 (0.11, 0.32)
Vision (m)				
4-		ref	ref	ref
1-		2.14 (1.29, 3.55)	2.35 (1.45, 3.82)	2.22 (1.45, 3.39)
0-			2.38 (1.24, 4.56)	3.27 (1.86, 5.78)
Hearing impairment				
0	ref	ref	ref	ref
Moderate	1.99 (1.33, 2.98)	3.65 (2.18, 6.12)	1.93 (1.19, 3.11)	2.36 (1.55, 3.60)
Somatosensory dysfunction				
0	ref	ref	ref	ref
Mild	1.97 (1.31, 2.95)		3.19 (1.87, 5.44)	2.39 (1.54, 3.71)
Moderate	11.58 (6.54, 20.49)	7.55 (3.85, 14.82)	15.47 (8.00, 29.92)	11.44 (6.15, 21.28)
Severe		38.92 (7.03, 215.62)	18.18 (3.45, 95.85)	48.14 (7.61, 304.40)
Hypertension				
Cerebrovascular disease		2.22 (1.19, 4.16)		2.76 (1.60, 4.76)
Linguistic incompetence		2.71 (1.26, 5.83)	2.22 (1.01, 4.88)	
Chronic bronchitis	2.03 (1.02, 4.02)			
Hemorrhoids	0.30 (0.11, 0.79)			

Non-significant factors were not presented. Significance level is 0.05

**Table 10:** Factors associated with impairments in individual items of balance

Variable	I 1	I 2	I 3	I 4	II 5	II 6	II 7	II 8	III 9	III 10	III 11	III 12	III 13	III 14	III 15	III 16
Gender																
Age (yrs)																
60-																
65-							**+**	**+**								
70-				**+**			**+**	**+**								
75-		**+**	**+**	**+**	**+**	**+**	**+**	**+**	**+**	**+**	**+**	**+**	**+**			**+**
80-		**+**	**+**	**+**	**+**	**+**	**+**	**+**	**+**	**+**	**+**	**+**	**+**	**+**	**+**	**+**
85–97		**+**	**+**	**+**	**+**	**+**	**+**	**+**	**+**	**+**	**+**	**+**	**+**	**+**	**+**	**+**
Education																
Elementary school or below	**+**		**+**	**+**							**+**					
Middle or high school				**+**												
College or above																
Income		**-**	**-**		**-**					**-**		**-**	**-**			
BMI (kg/m^2^)																
Underweight (< 18.5)			**+**		**+**											
Overweight or obesity [24.0-)					**+**	**+**	**+**	**+**	**+**	**+**	**+**	**+**	**+**		**+**	**+**
Normal [18.5–24.0)																
Exercise (times/week, > 10 min/time)																
0-																
1-	**-**	**-**		**-**				**-**				**-**	**-**		**-**	**-**
4-	**-**	**-**	**-**	**-**	**-**			**-**			**-**	**-**	**-**		**-**	**-**
7-	**-**	**-**	**-**	**-**	**-**		**-**	**-**	**-**	**-**	**-**	**-**	**-**	**-**	**-**	**-**
Smoking																
Vision (m)																
4-																
1-				**+**	**+**	**+**		**+**	**+**	**+**	**+**	**+**		**+**	**+**	**+**
0-		**+**	**+**	**+**	**+**		**+**	**+**	**+**	**+**	**+**				**+**	**+**
Eye diseases																
Hearing impairment																
0																
Moderate	**+**	**+**	**+**		**+**	**+**	**+**	**+**	**+**	**+**	**+**	**+**	**+**	**+**	**+**	**+**
Severe	**+**															
Profound																
Somatosensory dysfunction																
0																
Mild				**+**	**+**	**+**	**+**	**+**		**+**		**+**	**+**		**+**	**+**
Moderate	**+**	**+**	**+**	**+**	**+**	**+**	**+**	**+**	**+**	**+**	**+**	**+**	**+**	**+**	**+**	**+**
Severe	**+**	**+**	**+**	**+**	**+**	**+**	**+**		**+**	**+**	**+**	**+**		**+**	**+**	**+**
Hypotension								**+**								
Hypertension	**+**									**+**						
Hyperlipidemia																
Cardiovascular disease																
Cerebrovascular disease					**+**	**+**	**+**	**+**		**+**				**+**		**+**
Linguistic incompetence									**+**			**+**		**+**	**+**	
Varicose veins																
Diabetes																
Chronic bronchitis				**+**									**+**			
Asthma	**+**															
Arthritis																
Osteoporosis																
Intervertebral disc herniation																
Hemorrhoids													**-**			
Prostate hypertrophy																

+ OR > 1, positively associated with balance impairments, potential risk factors for balance impairments. - OR < 1, negatively associated with balance impairments, potential protective factors for balance impairments. Non-significant factors were not presented. Significance level is 0.05

Age was positively correlated with impairments in nearly all the items. Except the performance in standing with feet together (item I 1), age was significantly related to impairments in the remaining 15 items (ORs from 1.72 to 9.35, Tables 7, 8 and 9).

Relative to normal BMI, overweight and obesity was significantly related with elevated risks of impairments in all items in postural stability (ORs from 1.64 to 1.93, Table 8). Except the walk path (item III 14), overweight and obesity was significantly related with elevated risks of impairments in the remaining 7 items in dynamic balance (ORs from 1.35 to 2.96, Table 9).

Distinct vision lesion (only able for less than 1 m, 0-) was positively significantly associated with performance impairments in standing with one foot in front (item I 2, OR = 1.79), standing with eye closed (item I 3, OR = 2.36), and standing on one leg (item I 4, OR = 1.79) in static balance domain (Table 7).

Vision lesions (1- and 0-) were positively significantly associated with performance impairments in all 4 items (ORs from 1.52 to 2.00) in postural stability (Table 8). Except step continuity (item III 13), vision lesions (1- and 0-) were positively significantly associated with performance impairments in the remaining 7 items (ORs from 1.80 to 3.27) in dynamic balance (Table 9).

Somesthesis dysfunction was significantly associated with elevated risks of performance impairments in all items in static balance (ORs from 1.81 to 21.53, Table 7), postural stability (ORs from 2.31 to 170.76, Table 8), and dynamic balance (OR, from 1.62 to 48.14, Table 9). And aggravating dysfunction was associated with increased risks of impairments.

Cerebrovascular disease was significantly associated with elevated risks of performance impairments in all items in postural stability (ORs from 1.71 to 2.37, Table 8), and in step height, walk path, and turning while walking (items III 10, III 14, and III 16) as well, with ORs of 1.89, 2.22, and 2.76, respectively (Table 9).

Regular exercise was significantly related to reduced risks of impairments in all the items of static balance (ORs from 0.27 to 0.59, Table 7), 3 items of postural stability (ORs, from 0.44 to 0.70, Table 8), and all items of dynamic balance (OR, from 0.19 to 0.60, Table 9).

## Discussion

The present study provided factors associated with balance performance in the community-dwelling elderly. Age, overweight and obesity, exercise, vision, hearing, somesthesia, and cerebrovascular disease were dominant factors associated with impairments in overall balance, all domains, and most individual items.

### Age and gender

In the elderly, increasing age brought about increased risks of balance impairments in all aspects with slightly varying strengths of associations. In contrast the significant role of age, gender did not play evident role.

In static balance, age had the strongest strength of association with standing on one leg (item I 4) compared to strengths with standing with feet together with eyes opened and closed (item I 1 and item I 3). These results confirmed differences in declining trends between items. Previous findings indicated abilities of standing on one leg decreased with increasing age more significantly than abilities of standing with feet together with eyes opened and closed in the elderly [25].

The study revealed all 4 items of postural stability were associated with age. And age had higher strengths of associations with transitions from standing to sitting (item II 5) and standing to squatting (item II 7) than transitions from sitting to standing (item II 6) and squatting to standing (item II 8). Functional base of support (FBOS) was a measure of postural stability during forward and backward moving. It was reported that in community-dwelling elderly aged 60 to 91 years old, with increasing age, FBOS declined, subsequently, FBOS sway increased [26]. Decreased FBOS and increased FBOS sway could explain declined performances of postural stability with age. Physiologic decrements induced by aging in skeletal muscle power, strength, and mass contributed largely to deteriorations in balance performance. Major musculature of lower limb required for backward and forward movements were distinct [27]. Higher strengths of associations with transitions from standing to sitting and standing to squatting possibly hinted declined ability to perform backward movement might be at a greater extent than forward movement, which might be attributable to differential decrements in muscle functions by aging.

Dynamic balance performance depended on integration of muscles, skeletons, neural inputs and processing, cardiorespiratory capacity, and metabolism. During aging, functions in these aspects would by definition deteriorate, which would lead to corresponding impairments in task performance [27–33]. In the present study performances of all individual items in dynamic balance declined independently with increased age, the highest strength of association was found with step continuity (item III 13). Decremented performances indicated functional deteriorations in the elderly. Relatively, step continuity reflected comprehensive performance abilities of combined items, dysfunctions in any involved aspect would result in its impairment.

Among 3 domains, age had the least association with static balance and the strongest strength of association with postural stability. In contrast to static balance, postural stability and dynamic balance subjected to effects of physiologic decrements, function deteriorations, life styles, and exercises more sensitively [27, 34].

### Somesthesia

In general, somatosensory dysfunction showed strong association with balance impairments in all aspects, and somatosensory dysfunction showed stronger association with postural stability and dynamic balance than static balance. The findings were expected. Effective spatial orientation and balance required the integration of proprioceptive, vestibular, and vision. Associations of somesthesia with balance performance were well established. With increasing age, somatosensory deterioration was developed followed by increased body sway, decreased flexibility of the ankle-hip head axis, greater frailty, worse functional capacity, and higher fall prevalence [35–37]. Stronger association with postural stability and dynamic balance might be attributable to more profound contributions of physiologic decrements and function deteriorations [34].

### Vision and hearing

In regard to static balance performance, poor vision did not affect capacities of standings with feet together with eyes opened (Item I 1 and item I 2), however, poor vision was related to impairments in standing with feet together with eyes closed (Item I 3) and standing on one leg (Item I 4). A recent study in young and middled-aged adults reported that with normal vision, adults performed static balance better with eyes open than with eyes closed. And adults with visual impairment performed worse on standing on one leg than adults without visual impairment [38]. These results were in agreement with our findings in the elderly. Another study addressed the fact that participants with worse vision had higher failure rates of maintaining balance on foam surface [39]. These results suggested vision was one of the elements responsible for maintaining static balance though accurate vision might be less determined for maintaining upright position with feet together with eyes opened on firm surface.

As for postural stability and dynamic balance performances, poor vision was involved in their performance impairments of nearly all items. Valid visual information was vital for postural control and movement process and visual inputs could be relied heavily for certain population. Vision deficit could slow visual perception response, increased oscillations, decreased flexibility, consequently, influenced the ability of dynamic postural control and reduced gait velocity [10, 35, 40, 41].

Unexpectedly, the present study found that moderate hearing impairment increased risks of impairments in overall balance, all 3 domains, and 15 out of 16 items. While severe hearing impairment affected standing with feet together with eyes opened (Item I 1) only and profound hearing impairment did not show any effect on balance performance. There might be a potential that the elderly with severe and profound hearing impairments presented more caution and concentration during performing. The mechanisms need to be explored.

### Exercise

The exercise was independently associated with balance performances in all aspects. Regular exercise, longer than 10 min per time and the minimum 1 time per week, was related to reduced risks of balance impairments. Our findings confirmed and extended previous results. A large body of evidence showed exercises, of varying frequencies, duration, and intensities, were effective for improved balance performance in the elderly, and over the long run, were preventive for fall occurrence and fall-related injuries.

For example, in the elderly aged 60 years over, 6 weeks of strength exercise and balance exercise increased maximal strength of lower leg muscles, postural stability, and dynamic balance performances [42]. In the community-dwelling elderly aged 65 years over, 20 weeks of light-to-moderate intensity-oriented exercise intervention with three times per week improved overall functional fitness such as muscle resistance, hand grip strength, cardiorespiratory capacity, agility and balance, and flexibility [43]. In the elderly 65 years over, following 12 months of exercise program consisted of a warm-up, muscle strength, and cool-down exercises, grip strength and multiple motor functions were improved [44]. Multivariate logistic regression analysis revealed, every 60-min increase in light physical activity was associated with higher odds of good static balance [45]. In the elderly residing in Sydney, Australia, following 6 months of designed exercises to improve balance, coordination, aerobic capacity, and muscle strength, postural sways on the floor with eyes open and eyes closed and coordinated stability were improved. And following 12 months of designed exercises, rate of fall was lower than the controls [46]. Another study in the women elderly in Sydney, Australia reported, a 12 month program of regular exercise improved performances in leg muscle strength, reaction time, neuromuscular control, and body sway, further, reduced balance-related falls [47].

### Income and education

Associations between higher income and reduced risks of balance impairments were observed. The influences might be through effects on nutrition, exercise behavior, life style, and medical service. Within a certain range, the income was positively correlated to nutrition status, increased income partly ensured more balanced diets followed by better nutrient compositions. Lower income might be related to nutritional deficiency, and nutritional supplementation could be associated with greater muscle strength, better physical performance, and reduced fall occurrences [48, 49]. Moreover, income level was positively correlated with self-efficacy for exercise behavior [50]. Furthermore, increased income might confer access to higher quality of medical services.

Likewise, lower education level contributed to higher risks of balance impairments. In the present study, univariate analysis suggested lower education level (elementary school or below) were associated with higher risks of impairments in all aspects of overall balance performance, 3 domains, and 16 items.

### Potential interventions and preventions

Findings of the present study provided evidence on potentially effective intervention and prevention measures. For example, with increases in age, risks of impairments in all aspect of balance elevated gradually. Therefore, measures could be taken in all the elderly at the earliest possible. Measures could be taken to control overweight and obesity. Weekly exercise of 4 times or more could be recommended to reduce risks of impairments in all 3 domains and overall balance performance. Improvements in vision would be helpful. Improvements in somesthesia would be strikingly effective.

### Data collection and limitations

Demographic information and information on health status were selected based on literatures and previous studies of our research group. Inclusion criteria are as below. (1) There was literature evidence of associations with balance. (2) There were inconsistent results of associations with balance, which means further exploration is needed. (3) In theories on biology and medicine, there were potentials that the factors might affect balance performances.

In the association analyses, only exercise frequency was applied. Due to the incompleteness of detailed information on exercise mode, intensity, and duration, these variables and integrated indicators (for example, the metabolic equivalent for task, MET), could not be quantified thus related association analyses could not be conducted. Thereafter explorations for contributions of comprehensive exercise to balance performance are warranted in details with appropriate designs.

Contributions of occupations were not analyzed in the present study. Participants were the elderly, most of them were retired and their occupations prior to retirements were not collected, which was a flaw in the design. In the future, optimized design with information on detailed occupations together with corresponding durations and ceases might indicate contributions of occupation alone, occupations in combination, and their confounding effects.

As it was a cross-sectional study, causal relationships could not be established in this study.

## Conclusions

In the elderly, age, overweight and obesity, exercise, vision, hearing, somesthesia, and cerebrovascular disease were dominant factors associated with balance impairments. Increasing age brought about increased risks of balance impairments in all aspects measured. Gender did not play evident role. Overweight or obesity increased impairments in overall balance, postural stability, and dynamic balance. Regular exercise was independently associated with better balance performances in all aspects. Somatosensory dysfunction was associated with increased risks of balance impairments in all aspects. Poor vision deteriorated performances on overall balance, postural stability, and dynamic balance substantially.

### Electronic supplementary material

Below is the link to the electronic supplementary material.

**Table S1** Univariate logistic regression analysis on balance. **Table S2** Univariate logistic regression analysis on static balance. **Table S3** Univariate logistic regression analysis on postural stability. **Table S4** Univariate logistic regression analysis on dynamic balance. **Table S5** Univariate logistic regression analysis on individual items of static balance. **Table S6** Univariate logistic regression analysis on individual items of postural stability. **Table S7** Univariate logistic regression analysis on individual items of dynamic balance

## Data Availability

The data used in the study are available from the corresponding author on reasonable request.
