# Supplementary material for: Factors associated with balance impairments in the community-dwelling elderly in urban China
Source: BMC Geriatr. 2023 Sep 7;23:545. doi: 10.1186/s12877-023-04219-z (PMC10486131; doi:10.1186/s12877-023-04219-z)
Supplement: Supplementary file 1 — Table S1 Univariate logistic regression analysis on balance. Table S2 Univariate logistic regression analysis on static balance. Table S3 Univariate logistic regression analysis on postural stability. Table S4 Univariate logistic regression analysis on dynamic balance. Table S5 Univariate logistic regression analysis on individual items of static balance. Table S6 Univariate logistic regression analysis on individual items of postural stability. Table S7 Univariate logistic regression analysis on individual items of dynamic balance [file 12877_2023_4219_MOESM1_ESM.docx]

Table S1 Univariate logistic regression analysis on balance

| Variable | β | SE | Waldχ^2^ | *P* | OR | 95% CI |
| --- | --- | --- | --- | --- | --- | --- |
| Gender | 0.037 | 0.050 | 0.530 | 0.466 | 1.04 | (0.94, 1.14) |
| Age (yrs) |  |  |  |  |  |  |
| 60- | ref |  |  |  |  |  |
| 65- | 0.271 | 0.074 | 13.378 | <0.001 | 1.31 | (1.13, 1.52) |
| 70- | 0.418 | 0.080 | 27.512 | <0.001 | 1.52 | (1.30, 1.78) |
| 75- | 0.796 | 0.085 | 86.694 | <0.001 | 2.22 | (1.87, 2.62) |
| 80- | 1.178 | 0.107 | 120.753 | <0.001 | 3.25 | (2.63, 4.01) |
| 85-97 | 1.628 | 0.140 | 134.809 | <0.001 | 5.09 | (3.87, 6.70) |
| Education |  |  |  |  |  |  |
| Elementary school or below | 0.646 | 0.091 | 50.015 | <0.001 | 1.91 | (1.59, 2.28) |
| Middle or high school | -0.001 | 0.054 | 0.001 | 0.980 | 1.00 | (0.90, 1.11) |
| College or above | ref |  |  |  |  |  |
| Income | -0.065 | 0.018 | 13.633 | <0.001 | 0.94 | (0.90, 0.97) |
| BMI (kg/m^2^) |  |  |  |  |  |  |
| Underweight (<18.5) | 0.374 | 0.145 | 6.618 | 0.010 | 1.45 | (1.09, 1.93) |
| Overweight or obesity [24.0-) | 0.218 | 0.053 | 17.154 | <0.001 | 1.24 | (1.12, 1.38) |
| Normal [18.5-24.0) | ref |  |  |  |  |  |
| Exercise (times/week, >10 min/time) |  |  |  |  |  |  |
| 0- | ref |  |  |  |  |  |
| 1- | -0.464 | 0.076 | 37.681 | <0.001 | 0.63 | (0.54, 0.73) |
| 4- | -0.830 | 0.093 | 80.020 | <0.001 | 0.44 | (0.36, 0.52) |
| 7- | -0.765 | 0.068 | 128.159 | <0.001 | 0.47 | (0.41, 0.53) |
| Smoking | 0.061 | 0.062 | 0.958 | 0.328 | 1.06 | (0.94, 1.20) |
| Vision (m) |  |  |  |  |  |  |
| 4- | ref |  |  |  |  |  |
| 1- | 0.570 | 0.060 | 89.120 | <0.001 | 1.77 | (1.57, 1.99) |
| 0- | 0.937 | 0.101 | 85.906 | <0.001 | 2.55 | (2.09, 3.11) |
| Eye diseases | 0.240 | 0.089 | 7.270 | 0.007 | 1.27 | (1.07, 1.51) |
| Hearing impairment |  |  |  |  |  |  |
| 0 | ref |  |  |  |  |  |
| Mild | 0.866 | 0.071 | 148.936 | <0.001 | 2.38 | (2.07, 2.73) |
| Moderate | 1.474 | 0.186 | 62.580 | <0.001 | 4.37 | (3.03, 6.29) |
| Severe | 1.148 | 0.411 | 7.796 | 0.005 | 3.15 | (1.41, 7.06) |
| Somatosensory dysfunction |  |  |  |  |  |  |
| 0 | ref |  |  |  |  |  |
| Mild | 0.781 | 0.062 | 159.809 | <0.001 | 2.18 | (1.94, 2.47) |
| Moderate | 2.190 | 0.172 | 162.280 | <0.001 | 8.93 | (6.38, 12.51) |
| Severe | 3.032 | 0.725 | 17.499 | <0.001 | 20.74 | (5.01, 85.83) |
| Hypotension | 0.233 | 0.235 | 0.985 | 0.321 | 1.26 | (0.80, 2.00) |
| Hypertension | 0.237 | 0.051 | 21.961 | <0.001 | 1.27 | (1.15, 1.40) |
| Hyperlipidemia | 0.179 | 0.076 | 5.586 | 0.018 | 1.20 | (1.03, 1.39) |
| Cardiovascular disease | 0.387 | 0.073 | 28.023 | <0.001 | 1.47 | (1.28, 1.70) |
| Cerebrovascular disease | 0.665 | 0.105 | 39.750 | <0.001 | 1.94 | (1.58, 2.39) |
| Linguistic incompetence | 1.204 | 0.185 | 42.488 | <0.001 | 3.34 | (2.32, 4.79) |
| Varicose veins | 0.283 | 0.156 | 3.279 | 0.070 | 1.33 | (0.98, 1.80) |
| Diabetes | 0.157 | 0.076 | 4.261 | 0.039 | 1.17 | (1.01, 1.36) |
| Chronic bronchitis | 0.273 | 0.120 | 5.118 | 0.024 | 1.31 | (1.04, 1.66) |
| Asthma | 0.631 | 0.232 | 7.398 | 0.007 | 1.88 | (1.19, 2.96) |
| Arthritis | 0.155 | 0.082 | 3.516 | 0.061 | 1.17 | (0.99, 1.37) |
| Osteoporosis | 0.257 | 0.086 | 9.008 | 0.003 | 1.29 | (1.09, 1.53) |
| Intervertebral disc herniation | 0.081 | 0.113 | 0.514 | 0.473 | 1.08 | (0.87, 1.35) |
| Hemorrhoids | -0.013 | 0.105 | 0.016 | 0.901 | 0.99 | (0.80, 1.21) |
| Prostate hypertrophy | 0.196 | 0.121 | 2.604 | 0.107 | 1.22 | (0.96, 1.54) |

Table S2 Univariate logistic regression analysis on static balance

| Variable | β | SE | Waldχ^2^ | *P* | OR | 95% CI |
| --- | --- | --- | --- | --- | --- | --- |
| Gender | 0.085 | 0.049 | 3.011 | 0.083 | 1.09 | (0.99, 1.20) |
| Age (yrs) |  |  |  |  |  |  |
| 60- | ref |  |  |  |  |  |
| 65- | 0.155 | 0.070 | 4.871 | 0.027 | 1.17 | (1.02, 1.34) |
| 70- | 0.344 | 0.076 | 20.658 | <0.001 | 1.41 | (1.22, 1.64) |
| 75- | 0.628 | 0.081 | 59.459 | <0.001 | 1.87 | (1.60, 2.20) |
| 80- | 0.866 | 0.101 | 73.775 | <0.001 | 2.38 | (1.95, 2.90) |
| 85-97 | 1.197 | 0.127 | 88.647 | <0.001 | 3.31 | (2.58, 4.25) |
| Education |  |  |  |  |  |  |
| Elementary school or below | 0.676 | 0.089 | 58.026 | <0.001 | 1.97 | (1.65, 2.34) |
| Middle or high school | 0.095 | 0.053 | 3.228 | 0.072 | 1.10 | (0.99, 1.22) |
| College or above | ref |  |  |  |  |  |
| Income | -0.061 | 0.017 | 12.692 | <0.001 | 0.94 | (0.91, 0.97) |
| BMI (kg/m^2^) |  |  |  |  |  |  |
| Underweight (<18.5) | 0.403 | 0.142 | 8.019 | 0.005 | 1.50 | (1.13, 1.98) |
| Overweight or obesity [24.0-) | 0.121 | 0.051 | 5.600 | 0.018 | 1.13 | (1.02, 1.25) |
| Normal [18.5-24.0) | ref |  |  |  |  |  |
| Exercise (times/week, >10 min/time) |  |  |  |  |  |  |
| 0- | ref |  |  |  |  |  |
| 1- | -0.478 | 0.074 | 42.177 | <0.001 | 0.62 | (0.54, 0.72) |
| 4- | -0.782 | 0.090 | 75.119 | <0.001 | 0.46 | (0.38, 0.55) |
| 7- | -0.621 | 0.065 | 91.575 | <0.001 | 0.54 | (0.47, 0.61) |
| Smoking | -0.021 | 0.061 | 0.116 | 0.733 | 0.98 | (0.87, 1.10) |
| Vision (m) |  |  |  |  |  |  |
| 4- | ref |  |  |  |  |  |
| 1- | 0.339 | 0.058 | 34.022 | <0.001 | 1.40 | (1.25, 1.57) |
| 0- | 0.727 | 0.096 | 56.896 | <0.001 | 2.07 | (1.71, 2.50) |
| Eye diseases | 0.246 | 0.087 | 8.007 | 0.005 | 1.28 | (1.08, 1.52) |
| Hearing impairment |  |  |  |  |  |  |
| 0 | ref |  |  |  |  |  |
| Mild | 0.549 | 0.067 | 67.548 | <0.001 | 1.73 | (1.52, 1.97) |
| Moderate | 1.153 | 0.170 | 45.974 | <0.001 | 3.17 | (2.27, 4.42) |
| Severe | 0.669 | 0.373 | 3.223 | 0.073 | 1.95 | (0.94, 4.05) |
| Somatosensory dysfunction |  |  |  |  |  |  |
| 0 | ref |  |  |  |  |  |
| Mild | 0.442 | 0.058 | 57.635 | <0.001 | 1.56 | (1.39, 1.74) |
| Moderate | 1.278 | 0.133 | 92.125 | <0.001 | 3.59 | (2.76, 4.66) |
| Severe | 2.067 | 0.512 | 16.312 | <0.001 | 7.90 | (2.90, 21.56) |
| Hypotension | -0.137 | 0.232 | 0.350 | 0.554 | 0.87 | (0.55, 1.37) |
| Hypertension | 0.248 | 0.049 | 25.218 | <0.001 | 1.28 | (1.16, 1.41) |
| Hyperlipidemia | 0.148 | 0.074 | 4.001 | 0.045 | 1.16 | (1.00, 1.34) |
| Cardiovascular disease | 0.224 | 0.071 | 10.073 | 0.002 | 1.25 | (1.09, 1.44) |
| Cerebrovascular disease | 0.456 | 0.100 | 20.795 | <0.001 | 1.58 | (1.30, 1.92) |
| Linguistic incompetence | 0.958 | 0.167 | 32.704 | <0.001 | 2.61 | (1.88, 3.62) |
| Varicose veins | -0.120 | 0.153 | 0.613 | 0.434 | 0.89 | (0.66, 1.20) |
| Diabetes | 0.079 | 0.074 | 1.144 | 0.285 | 1.08 | (0.94, 1.25) |
| Chronic bronchitis | 0.183 | 0.117 | 2.441 | 0.118 | 1.20 | (0.95, 1.51) |
| Asthma | 0.549 | 0.223 | 6.086 | 0.014 | 1.73 | (1.12, 2.68) |
| Arthritis | 0.054 | 0.080 | 0.448 | 0.503 | 1.06 | (0.90, 1.24) |
| Osteoporosis | 0.121 | 0.083 | 2.107 | 0.147 | 1.13 | (0.96, 1.33) |
| Intervertebral disc herniation | 0.078 | 0.110 | 0.499 | 0.480 | 1.08 | (0.87, 1.34) |
| Hemorrhoids | 0.039 | 0.102 | 0.146 | 0.702 | 1.04 | (0.85, 1.27) |
| Prostate hypertrophy | 0.107 | 0.118 | 0.820 | 0.365 | 1.11 | (0.88, 1.40) |

Table S3 Univariate logistic regression analysis on postural stability

| Variable | β | SE | Waldχ^2^ | *P* | OR | 95% CI |
| --- | --- | --- | --- | --- | --- | --- |
| Gender | 0.071 | 0.048 | 2.203 | 0.138 | 1.07 | (0.98, 1.18) |
| Age (yrs) |  |  |  |  |  |  |
| 60- | ref |  |  |  |  |  |
| 65- | 0.318 | 0.070 | 20.763 | <0.001 | 1.37 | (1.20, 1.58) |
| 70- | 0.437 | 0.075 | 33.484 | <0.001 | 1.55 | (1.33, 1.79) |
| 75- | 0.820 | 0.081 | 103.035 | <0.001 | 2.27 | (1.94, 2.66) |
| 80- | 1.031 | 0.098 | 110.561 | <0.001 | 2.80 | (2.31, 3.40) |
| 85-97 | 1.502 | 0.122 | 152.390 | <0.001 | 4.49 | (3.54, 5.70) |
| Education |  |  |  |  |  |  |
| Elementary school or below | 0.483 | 0.083 | 33.554 | <0.001 | 1.62 | (1.38, 1.91) |
| Middle or high school | -0.056 | 0.051 | 1.193 | 0.275 | 0.95 | (0.86, 1.05) |
| College or above | ref |  |  |  |  |  |
| Income | -0.036 | 0.017 | 4.652 | 0.031 | 0.96 | (0.93, 1.00) |
| BMI (kg/m^2^) |  |  |  |  |  |  |
| Underweight (<18.5) | 0.415 | 0.135 | 9.377 | 0.002 | 1.51 | (1.16, 1.97) |
| Overweight or obesity [24.0-) | 0.231 | 0.050 | 21.602 | <0.001 | 1.26 | (1.14, 1.39) |
| Normal [18.5-24.0) | ref |  |  |  |  |  |
| Exercise (times/week, >10 min/time) |  |  |  |  |  |  |
| 0- | ref |  |  |  |  |  |
| 1- | -0.391 | 0.071 | 30.598 | <0.001 | 0.68 | (0.59, 0.78) |
| 4- | -0.624 | 0.086 | 52.732 | <0.001 | 0.54 | (0.45, 0.63) |
| 7- | -0.693 | 0.063 | 119.236 | <0.001 | 0.50 | (0.44, 0.57) |
| Smoking | 0.012 | 0.059 | 0.042 | 0.837 | 1.01 | (0.90, 1.14) |
| Vision (m) |  |  |  |  |  |  |
| 4- | ref |  |  |  |  |  |
| 1- | 0.450 | 0.057 | 62.989 | <0.001 | 1.57 | (1.40, 1.75) |
| 0- | 0.738 | 0.091 | 65.955 | <0.001 | 2.09 | (1.75, 2.50) |
| Eye diseases | 0.168 | 0.084 | 4.022 | 0.045 | 1.18 | (1.00, 1.39) |
| Hearing impairment |  |  |  |  |  |  |
| 0 | ref |  |  |  |  |  |
| Mild | 0.711 | 0.065 | 118.186 | <0.001 | 2.04 | (1.79, 2.31) |
| Moderate | 1.242 | 0.153 | 65.954 | <0.001 | 3.46 | (2.57, 4.67) |
| Severe | 0.829 | 0.349 | 5.634 | 0.018 | 2.29 | (1.16, 4.54) |
| Somatosensory dysfunction |  |  |  |  |  |  |
| 0 | ref |  |  |  |  |  |
| Mild | 0.781 | 0.059 | 174.275 | <0.001 | 2.18 | (1.94, 2.45) |
| Moderate | 1.682 | 0.126 | 179.021 | <0.001 | 5.38 | (4.20, 6.88) |
| Severe | 2.423 | 0.414 | 34.192 | <0.001 | 11.28 | (5.01, 25.41) |
| Hypotension | 0.309 | 0.219 | 1.993 | 0.158 | 1.36 | (0.89, 2.09) |
| Hypertension | 0.219 | 0.048 | 20.936 | <0.001 | 1.25 | (1.13, 1.37) |
| Hyperlipidemia | 0.205 | 0.071 | 8.259 | 0.004 | 1.23 | (1.07, 1.41) |
| Cardiovascular disease | 0.334 | 0.068 | 24.046 | <0.001 | 1.40 | (1.22, 1.60) |
| Cerebrovascular disease | 0.674 | 0.096 | 49.363 | <0.001 | 1.96 | (1.63, 2.37) |
| Linguistic incompetence | 1.110 | 0.154 | 52.076 | <0.001 | 3.03 | (2.24, 4.10) |
| Varicose veins | 0.242 | 0.146 | 2.752 | 0.097 | 1.27 | (0.96, 1.69) |
| Diabetes | 0.162 | 0.071 | 5.159 | 0.023 | 1.18 | (1.02, 1.35) |
| Chronic bronchitis | 0.295 | 0.112 | 6.903 | 0.009 | 1.34 | (1.08, 1.67) |
| Asthma | 0.551 | 0.208 | 7.036 | 0.008 | 1.73 | (1.15, 2.60) |
| Arthritis | 0.212 | 0.077 | 7.470 | 0.006 | 1.24 | (1.06, 1.44) |
| Osteoporosis | 0.323 | 0.080 | 16.308 | <0.001 | 1.38 | (1.18, 1.62) |
| Intervertebral disc herniation | 0.081 | 0.106 | 0.580 | 0.446 | 1.08 | (0.88, 1.33) |
| Hemorrhoids | -0.030 | 0.100 | 0.092 | 0.761 | 0.97 | (0.80, 1.18) |
| Prostate hypertrophy | 0.192 | 0.114 | 2.853 | 0.091 | 1.21 | (0.97, 1.51) |

Table S4 Univariate logistic regression analysis on dynamic balance

| Variable | β | SE | Waldχ^2^ | *P* | OR | 95% CI |
| --- | --- | --- | --- | --- | --- | --- |
| Gender | 0.038 | 0.049 | 0.594 | 0.441 | 1.04 | (0.94, 1.14) |
| Age (yrs) |  |  |  |  |  |  |
| 60- | ref |  |  |  |  |  |
| 65- | 0.214 | 0.070 | 9.228 | 0.002 | 1.24 | (1.08, 1.42) |
| 70- | 0.268 | 0.076 | 12.328 | <0.001 | 1.31 | (1.13, 1.52) |
| 75- | 0.621 | 0.081 | 58.757 | <0.001 | 1.86 | (1.59, 2.18) |
| 80- | 0.961 | 0.100 | 92.355 | <0.001 | 2.61 | (2.15, 3.18) |
| 85-97 | 1.371 | 0.127 | 116.935 | <0.001 | 3.94 | (3.07, 5.05) |
| Education |  |  |  |  |  |  |
| Elementary school or below | 0.593 | 0.087 | 46.919 | <0.001 | 1.81 | (1.53, 2.15) |
| Middle or high school | 0.045 | 0.052 | 0.733 | 0.392 | 1.05 | (0.94, 1.16) |
| College or above | ref |  |  |  |  |  |
| Income | -0.060 | 0.017 | 12.173 | <0.001 | 0.94 | (0.91, 0.97) |
| BMI (kg/m^2^) |  |  |  |  |  |  |
| Underweight (<18.5) | 0.439 | 0.140 | 9.819 | 0.002 | 1.55 | (1.18, 2.04) |
| Overweight or obesity [24.0-) | 0.203 | 0.051 | 15.792 | <0.001 | 1.22 | (1.11, 1.35) |
| Normal [18.5-24.0) | ref |  |  |  |  |  |
| Exercise (times/week, >10 min/time) |  |  |  |  |  |  |
| 0- | ref |  |  |  |  |  |
| 1- | -0.361 | 0.072 | 25.053 | <0.001 | 0.70 | (0.61, 0.80) |
| 4- | -0.607 | 0.087 | 48.165 | <0.001 | 0.54 | (0.46, 0.65) |
| 7- | -0.713 | 0.065 | 121.205 | <0.001 | 0.49 | (0.43, 0.56) |
| Smoking | 0.060 | 0.060 | 0.990 | 0.320 | 1.06 | (0.94, 1.19) |
| Vision (m) |  |  |  |  |  |  |
| 4- | ref |  |  |  |  |  |
| 1- | 0.533 | 0.058 | 84.396 | <0.001 | 1.70 | (1.52, 1.91) |
| 0- | 0.811 | 0.094 | 73.872 | <0.001 | 2.25 | (1.87, 2.71) |
| Eye diseases | 0.189 | 0.086 | 4.812 | 0.028 | 1.21 | (1.02, 1.43) |
| Hearing impairment |  |  |  |  |  |  |
| 0 | ref |  |  |  |  |  |
| Mild | 0.752 | 0.067 | 127.070 | <0.001 | 2.12 | (1.86, 2.42) |
| Moderate | 1.214 | 0.162 | 55.976 | <0.001 | 3.37 | (2.45, 4.63) |
| Severe | 1.049 | 0.377 | 7.722 | 0.005 | 2.85 | (1.36, 5.98) |
| Somatosensory dysfunction |  |  |  |  |  |  |
| 0 | ref |  |  |  |  |  |
| Mild | 0.653 | 0.058 | 125.390 | <0.001 | 1.92 | (1.71, 2.15) |
| Moderate | 1.765 | 0.139 | 161.644 | <0.001 | 5.84 | (4.45, 7.67) |
| Severe | 2.285 | 0.482 | 22.501 | <0.001 | 9.83 | (3.82, 25.26) |
| Hypotension | 0.217 | 0.226 | 0.921 | 0.337 | 1.24 | (0.80, 1.94) |
| Hypertension | 0.162 | 0.049 | 10.879 | 0.001 | 1.18 | (1.07, 1.29) |
| Hyperlipidemia | 0.067 | 0.073 | 0.833 | 0.362 | 1.07 | (0.93, 1.23) |
| Cardiovascular disease | 0.272 | 0.070 | 15.096 | <0.001 | 1.31 | (1.14, 1.51) |
| Cerebrovascular disease | 0.591 | 0.099 | 35.437 | <0.001 | 1.81 | (1.49, 2.19) |
| Linguistic incompetence | 1.238 | 0.172 | 51.673 | <0.001 | 3.45 | (2.46, 4.84) |
| Varicose veins | 0.256 | 0.150 | 2.906 | 0.088 | 1.29 | (0.96, 1.73) |
| Diabetes | 0.132 | 0.073 | 3.209 | 0.073 | 1.14 | (0.99, 1.32) |
| Chronic bronchitis | 0.193 | 0.116 | 2.763 | 0.096 | 1.21 | (0.97, 1.52) |
| Asthma | 0.347 | 0.214 | 2.640 | 0.104 | 1.42 | (0.93, 2.15) |
| Arthritis | 0.141 | 0.080 | 3.138 | 0.076 | 1.15 | (0.99, 1.35) |
| Osteoporosis | 0.180 | 0.082 | 4.765 | 0.029 | 1.20 | (1.02, 1.41) |
| Intervertebral disc herniation | 0.086 | 0.109 | 0.621 | 0.431 | 1.09 | (0.88, 1.35) |
| Hemorrhoids | -0.077 | 0.103 | 0.563 | 0.453 | 0.93 | (0.76, 1.13) |
| Prostate hypertrophy | 0.190 | 0.117 | 2.634 | 0.105 | 1.21 | (0.96, 1.52) |

Table S5 Univariate logistic regression analysis on individual items of static balance

| Variable | I 1 | I 2 | I 3 | I 4 |
| --- | --- | --- | --- | --- |
| Gender | 1.24 (0.90, 1.73) | 1.11 (0.81, 1.53) | 1.20 (0.91, 1.59) | 1.19 (0.97, 1.47) |
| Age (yrs) |  |  |  |  |
| 60- | ref | ref | ref | ref |
| 65- | 1.30 (0.73, 2.32) | 1.55 (0.86, 2.80) | 1.30 (0.76, 2.25) | 1.50 (1.05, 2.15) |
| 70- | 1.72 (0.95, 3.11) | 1.60 (0.85, 3.00) | 2.14 (1.25, 3.65) | 2.53 (1.76, 3.63) |
| 75- | 2.72 (1.53, 4.83) | 3.57 (2.01, 6.35) | 4.01 (2.40, 6.69) | 4.69 (3.26, 6.73) |
| 80- | 4.57 (2.50, 8.32) | 6.47 (3.57, 11.75) | 7.38 (4.32, 12.61) | 6.32 (4.18, 9.55) |
| 85-97 | 7.48 (3.99, 14.01) | 10.15 (5.42, 19.02) | 13.42 (7.60, 23.70) | 14.21 (8.72, 23.14) |
| Education |  |  |  |  |
| Elementary school or below | 3.49 (2.23, 5.44) | 4.01 (2.55, 6.30) | 4.24 (2.85, 6.30) | 3.83 (2.76, 5.33) |
| Middle or high school | 0.96 (0.66, 1.40) | 1.33 (0.92, 1.94) | 1.19 (0.86, 1.66) | 1.29 (1.02, 1.63) |
| College or above | ref | ref | ref | ref |
| Income | 0.89 (0.75, 1.04) | 0.78 (0.67, 0.91) | 0.77 (0.67, 0.88) | 0.82 (0.74, 0.90) |
| BMI (kg/m^2^) |  |  |  |  |
| Underweight (<18.5) | 1.33 (0.56, 3.18) | 3.01 (1.55, 5.87) | 3.37 (1.87, 6.07) | 2.35 (1.39, 3.96) |
| Overweight or obesity [24.0-) | 1.23 (0.88, 1.72) | 1.37 (0.99, 1.90) | 1.30 (0.97, 1.74) | 1.30 (1.05, 1.61) |
| Normal [18.5-24.0) | ref | ref | ref | ref |
| Exercise (times/week, >10 min/time) |  |  |  |  |
| 0- | ref | ref | ref | ref |
| 1- | 0.37 (0.24, 0.58) | 0.28 (0.18, 0.44) | 0.50 (0.35, 0.72) | 0.36 (0.27, 0.49) |
| 4- | 0.20 (0.10, 0.41) | 0.14 (0.07, 0.30) | 0.18 (0.10, 0.34) | 0.18 (0.12, 0.28) |
| 7- | 0.32 (0.22, 0.47) | 0.26 (0.18, 0.37) | 0.26 (0.19, 0.37) | 0.26 (0.20, 0.34) |
| Smoking | 0.98 (0.65, 1.46) | 0.89 (0.60, 1.33) | 0.92 (0.65, 1.31) | 1.00 (0.77, 1.29) |
| Vision (m) |  |  |  |  |
| 4- | ref | ref | ref | ref |
| 1- | 1.71 (1.17, 2.49) | 1.58 (1.09, 2.29) | 2.34 (1.71, 3.21) | 2.31 (1.83, 2.92) |
| 0- | 4.03 (2.56, 6.34) | 4.52 (2.93, 6.96) | 5.64 (3.79, 8.40) | 3.66 (2.59, 5.18) |
| Eye diseases | 1.90 (1.19, 3.05) | 1.85 (1.16, 2.94) | 1.89 (1.25, 2.86) | 1.43 (1.01, 2.01) |
| Hearing impairment |  |  |  |  |
| 0 | ref | ref | ref | ref |
| Mild | 2.78 (1.91, 4.05) | 2.89 (2.01, 4.14) | 4.26 (3.12, 5.82) | 3.09 (2.41, 3.98) |
| Moderate | 9.15 (5.03, 16.64) | 9.15 (5.07, 16.50) | 9.33 (5.27, 16.53) | 7.22 (4.10, 12.71) |
| Severe | 8.43 (2.07, 34.25) | 1.94 (0.24, 15.70) | 3.59 (0.74, 17.50) | 3.40 (0.91, 12.72) |
| Somatosensory dysfunction |  |  |  |  |
| 0 | ref | ref | ref | ref |
| Mild | 2.07 (1.40, 3.05) | 2.21 (1.51, 3.22) | 2.94 (2.14, 4.03) | 2.84 (2.25, 3.58) |
| Moderate | 12.23 (7.55, 19.81) | 14.81 (9.22, 23.79) | 12.67 (8.01, 20.03) | 9.86 (6.26, 15.51) |
| Severe | 45.18 (11.43, 178.50) | 43.84 (11.10, 173.12) | 32.92 (8.37, 129.38) | 19.71 (4.16, 93.41) |
| Hypotension | 0.52 (0.07, 3.90) | 0.49 (0.06, 3.62) | 1.17 (0.34, 3.97) | 0.65 (0.22, 1.93) |
| Hypertension | 2.04 (1.46, 2.85) | 1.84 (1.33, 2.53) | 1.38 (1.05, 1.83) | 1.68 (1.36, 2.06) |
| Hyperlipidemia | 1.49 (0.96, 2.31) | 1.43 (0.93, 2.20) | 1.32 (0.90, 1.95) | 1.32 (0.98, 1.77) |
| Cardiovascular disease | 1.49 (0.98, 2.27) | 1.49 (0.99, 2.24) | 1.47 (1.02, 2.11) | 1.81 (1.38, 2.38) |
| Cerebrovascular disease | 2.85 (1.77, 4.59) | 2.93 (1.85, 4.65) | 2.48 (1.61, 3.83) | 2.01 (1.39, 2.90) |
| Linguistic incompetence | 6.39 (3.54, 11.55) | 7.66 (4.32, 13.58) | 6.84 (3.92, 11.94) | 4.43 (2.56, 7.68) |
| Varicose veins | 1.21 (0.47, 3.09) | 0.87 (0.31, 2.45) | 0.63 (0.22, 1.76) | 0.63 (0.31, 1.30) |
| Diabetes | 1.02 (0.62, 1.66) | 1.30 (0.84, 2.03) | 1.13 (0.75, 1.69) | 1.21 (0.90, 1.63) |
| Chronic bronchitis | 1.12 (0.53, 2.36) | 1.04 (0.49, 2.18) | 0.96 (0.49, 1.88) | 2.03 (1.31, 3.13) |
| Asthma | 4.41 (1.82, 10.65) | 3.30 (1.31, 8.34) | 2.37 (0.94, 5.97) | 3.18 (1.47, 6.92) |
| Arthritis | 1.29 (0.79, 2.10) | 1.26 (0.78, 2.04) | 1.20 (0.78, 1.85) | 1.01 (0.72, 1.41) |
| Osteoporosis | 1.32 (0.80, 2.19) | 1.38 (0.85, 2.24) | 1.60 (1.06, 2.42) | 1.13 (0.80, 1.59) |
| Intervertebral disc herniation | 1.24 (0.64, 2.44) | 1.02 (0.50, 2.06) | 1.02 (0.55, 1.88) | 1.16 (0.74, 1.81) |
| Hemorrhoids | 1.56 (0.87, 2.80) | 1.44 (0.81, 2.58) | 1.39 (0.83, 2.34) | 0.76 (0.48, 1.20) |
| Prostate hypertrophy | 1.15 (0.55, 2.42) | 1.73 (0.92, 3.24) | 0.87 (0.43, 1.76) | 1.31 (0.82, 2.09) |

Table S6 Univariate logistic regression analysis on individual items of postural stability

| Variable | Ⅱ 5 | Ⅱ 6 | Ⅱ 7 | Ⅱ 8 |
| --- | --- | --- | --- | --- |
| Gender | 1.05 (0.81, 1.37) | 1.14 (0.89, 1.46) | 1.23 (1.00, 1.51) | 1.16 (0.95, 1.42) |
| Age (yrs) |  |  |  |  |
| 60- | ref | ref | ref | ref |
| 65- | 2.00 (1.14, 3.52) | 1.80 (1.11, 2.90) | 2.32 (1.58, 3.40) | 1.95 (1.39, 2.74) |
| 70- | 2.07 (1.14, 3.77) | 1.82 (1.09, 3.03) | 3.16 (2.13, 4.69) | 2.80 (1.97, 3.97) |
| 75- | 6.97 (4.08, 11.92) | 5.83 (3.67, 9.25) | 6.22 (4.20, 9.20) | 4.80 (3.36, 6.84) |
| 80- | 13.67 (7.81, 23.91) | 8.62 (5.23, 14.22) | 10.5 (6.78, 16.24) | 6.94 (4.63, 10.42) |
| 85-97 | 26.40 (14.50, 48.08) | 23.13 (13.41, 39.91) | 17.86 (10.77, 29.63) | 13.73 (8.45, 22.31) |
| Education |  |  |  |  |
| Elementary school or below | 3.99 (2.73, 5.81) | 3.28 (2.31, 4.67) | 2.16 (1.56, 2.99) | 1.95 (1.42, 2.69) |
| Middle or high school | 1.11 (0.82, 1.50) | 0.82 (0.62, 1.09) | 0.84 (0.67, 1.05) | 0.86 (0.69, 1.06) |
| College or above | ref | ref | ref | ref |
| Income | 0.81 (0.71, 0.92) | 0.86 (0.76, 0.97) | 0.90 (0.82, 1.00) | 0.90 (0.82, 0.99) |
| BMI (kg/m^2^) |  |  |  |  |
| Underweight (<18.5) | 3.28 (1.82, 5.90) | 2.93 (1.65, 5.20) | 1.81 (1.05, 3.13) | 1.95 (1.15, 3.32) |
| Overweight or obesity [24.0-) | 1.72 (1.31, 2.25) | 1.75 (1.36, 2.26) | 1.57 (1.27, 1.94) | 1.71 (1.40, 2.10) |
| Normal [18.5-24.0) | ref | ref | ref | ref |
| Exercise (times/week, >10 min/time) |  |  |  |  |
| 0- | ref | ref | ref | ref |
| 1- | 0.46 (0.33, 0.65) | 0.59 (0.43, 0.81) | 0.63 (0.48, 0.83) | 0.56 (0.42, 0.73) |
| 4- | 0.22 (0.13, 0.37) | 0.28 (0.18, 0.45) | 0.36 (0.25, 0.52) | 0.33 (0.23, 0.48) |
| 7- | 0.20 (0.14, 0.28) | 0.28 (0.21, 0.38) | 0.31 (0.24, 0.40) | 0.32 (0.25, 0.41) |
| Smoking | 1.14 (0.83, 1.57) | 1.04 (0.77, 1.40) | 0.89 (0.69, 1.15) | 1.06 (0.83, 1.35) |
| Vision (m) |  |  |  |  |
| 4- | ref | ref | ref | ref |
| 1- | 3.36 (2.50, 4.51) | 3.24 (2.46, 4.25) | 2.41 (1.90, 3.04) | 2.70 (2.16, 3.39) |
| 0- | 5.70 (3.84, 8.45) | 4.41 (3.00, 6.49) | 3.91 (2.76, 5.54) | 3.84 (2.72, 5.41) |
| Eye diseases | 1.33 (0.87, 2.05) | 1.24 (0.82, 1.86) | 1.37 (0.97, 1.94) | 1.31 (0.94, 1.83) |
| Hearing impairment |  |  |  |  |
| 0 | ref | ref | ref | ref |
| Mild | 5.31 (3.95, 7.15) | 5.73 (4.33, 7.58) | 3.88 (3.02, 4.99) | 3.97 (3.10, 5.08) |
| Moderate | 11.81 (6.72, 20.77) | 12.17 (6.93, 21.37) | 8.46 (4.77, 15.01) | 9.10 (5.01, 16.52) |
| Severe | 5.91 (1.46, 23.91) | 2.78 (0.57, 13.51) | 2.30 (0.57, 9.24) | 3.06 (0.82, 11.47) |
| Somatosensory dysfunction |  |  |  |  |
| 0 | ref | ref | ref | ref |
| Mild | 5.22 (3.79, 7.17) | 7.00 (5.20, 9.41) | 4.83 (3.81, 6.13) | 4.40 (3.50, 5.54) |
| Moderate | 46.35 (27.84, 77.17) | 43.88 (26.36, 73.07) | 31.54 (18.01, 55.23) | 26.43 (14.94, 46.75) |
| Severe | 161.88 (20.24, 1294.72) | 145.21 (18.18, 1159.97) | 58.98 (7.43, 468.25) |  |
| Hypotension | 1.42 (0.48, 4.21) | 1.98 (0.77, 5.06) | 1.40 (0.57, 3.42) | 3.54 (1.54, 8.12) |
| Hypertension | 1.64 (1.26, 2.14) | 1.60 (1.25, 2.04) | 1.45 (1.18, 1.78) | 1.47 (1.21, 1.80) |
| Hyperlipidemia | 1.53 (1.07, 2.19) | 1.70 (1.22, 2.36) | 1.65 (1.23, 2.20) | 1.58 (1.20, 2.10) |
| Cardiovascular disease | 1.94 (1.40, 2.69) | 2.29 (1.69, 3.09) | 2.08 (1.59, 2.72) | 2.24 (1.73, 2.92) |
| Cerebrovascular disease | 3.55 (2.40, 5.26) | 4.30 (2.96, 6.24) | 3.31 (2.31, 4.74) | 3.32 (2.32, 4.75) |
| Linguistic incompetence | 7.39 (4.26, 12.82) | 7.59 (4.37, 13.17) | 5.31 (3.04, 9.26) | 4.41 (2.53, 7.69) |
| Varicose veins | 2.25 (1.18, 4.26) | 2.04 (1.09, 3.80) | 1.52 (0.85, 2.74) | 1.64 (0.94, 2.89) |
| Diabetes | 1.30 (0.90, 1.88) | 1.39 (0.99, 1.96) | 1.69 (1.27, 2.26) | 1.59 (1.20, 2.11) |
| Chronic bronchitis | 1.71 (1.00, 2.91) | 2.20 (1.36, 3.55) | 1.69 (1.08, 2.64) | 1.72 (1.11, 2.65) |
| Asthma | 3.65 (1.61, 8.28) | 3.55 (1.60, 7.90) | 2.78 (1.27, 6.04) | 3.18 (1.46, 6.91) |
| Arthritis | 1.68 (1.15, 2.45) | 1.55 (1.08, 2.23) | 1.81 (1.33, 2.46) | 1.44 (1.06, 1.96) |
| Osteoporosis | 1.73 (1.18, 2.54) | 2.25 (1.59, 3.18) | 1.96 (1.43, 2.68) | 2.01 (1.48, 2.73) |
| Intervertebral disc herniation | 0.96 (0.53, 1.74) | 1.26 (0.75, 2.10) | 0.95 (0.60, 1.52) | 1.51 (1.00, 2.29) |
| Hemorrhoids | 0.87 (0.49, 1.55) | 0.90 (0.53, 1.53) | 0.82 (0.52, 1.29) | 1.09 (0.73, 1.64) |
| Prostate hypertrophy | 1.50 (0.86, 2.63) | 1.33 (0.77, 2.29) | 1.57 (1.00, 2.48) | 1.53 (0.98, 2.39) |

Table S7 Univariate logistic regression analysis on individual items of dynamic balance

| Variable | Ⅲ 9 | Ⅲ 10 | Ⅲ 11 | Ⅲ 12 |
| --- | --- | --- | --- | --- |
| Gender | 1.03 (0.75, 1.41) | 0.98 (0.74, 1.30) | 1.22 (0.96, 1.55) | 1.05 (0.78, 1.41) |
| Age (yrs) |  |  |  |  |
| 60- | ref | ref | ref | ref |
| 65- | 1.82 (0.91, 3.61) | 2.24 (1.25, 4.03) | 1.52 (1.00, 2.30) | 1.73 (0.92, 3.23) |
| 70- | 2.14 (1.05, 4.36) | 2.41 (1.31, 4.46) | 1.63 (1.05, 2.53) | 2.47 (1.32, 4.64) |
| 75- | 5.79 (3.04, 11.04) | 5.60 (3.16, 9.94) | 2.90 (1.89, 4.45) | 4.82 (2.65, 8.77) |
| 80- | 10.31 (5.30, 20.06) | 8.99 (4.93, 16.43) | 6.05 (3.85, 9.51) | 9.58 (5.18, 17.69) |
| 85-97 | 20.58 (10.39, 40.77) | 21.67 (11.64, 40.34) | 11.69 (7.08, 19.29) | 18.85 (9.97, 35.66) |
| Education |  |  |  |  |
| Elementary school or below | 3.07 (2.00, 4.73) | 3.07 (2.04, 4.62) | 3.60 (2.51, 5.16) | 3.26 (2.13, 4.99) |
| Middle or high school | 0.82 (0.57, 1.18) | 1.06 (0.77, 1.46) | 1.21 (0.92, 1.60) | 1.10 (0.78, 1.56) |
| College or above | ref | ref | ref | ref |
| Income | 0.87 (0.75, 1.02) | 0.84 (0.73, 0.97) | 0.86 (0.76, 0.97) | 0.83 (0.71, 0.96) |
| BMI (kg/m^2^) |  |  |  |  |
| Underweight (<18.5) | 3.95 (2.01, 7.77) | 2.35 (1.19, 4.65) | 1.55 (0.82, 2.91) | 2.46 (1.24, 4.87) |
| Overweight or obesity [24.0-) | 2.27 (1.63, 3.17) | 1.86 (1.39, 2.49) | 1.34 (1.05, 1.71) | 1.51 (1.11, 2.06) |
| Normal [18.5-24.0) | ref | ref | ref | ref |
| Exercise (times/week, >10 min/time) |  |  |  |  |
| 0- | ref | ref | ref | ref |
| 1- | 0.49 (0.33, 0.72) | 0.49 (0.34, 0.69) | 0.59 (0.43, 0.80) | 0.42 (0.29, 0.62) |
| 4- | 0.21 (0.11, 0.40) | 0.32 (0.19, 0.52) | 0.28 (0.17, 0.44) | 0.21 (0.12, 0.39) |
| 7- | 0.15 (0.10, 0.23) | 0.14 (0.09, 0.21) | 0.28 (0.21, 0.38) | 0.18 (0.12, 0.27) |
| Smoking | 1.33 (0.92, 1.92) | 1.26 (0.90, 1.76) | 0.87 (0.64, 1.18) | 1.14 (0.80, 1.63) |
| Vision (m) |  |  |  |  |
| 4- | ref | ref | ref | ref |
| 1- | 4.34 (3.00, 6.26) | 3.88 (2.81, 5.36) | 3.06 (2.34, 4.01) | 4.07 (2.91, 5.70) |
| 0- | 8.20 (5.21, 12.91) | 6.57 (4.33, 9.98) | 5.06 (3.49, 7.35) | 5.30 (3.37, 8.32) |
| Eye diseases | 2.44 (1.58, 3.77) | 1.68 (1.09, 2.58) | 1.53 (1.05, 2.25) | 1.26 (0.77, 2.06) |
| Hearing impairment |  |  |  |  |
| 0 | ref | ref | ref | ref |
| Mild | 5.10 (3.58, 7.27) | 6.28 (4.58, 8.62) | 3.75 (2.84, 4.95) | 6.49 (4.65, 9.06) |
| Moderate | 12.85 (7.09, 23.27) | 12.72 (7.15, 22.63) | 8.48 (4.87, 14.79) | 12.97 (7.21, 23.34) |
| Severe | 16.15 (4.25, 61.37) | 7.95 (1.96, 32.30) | 9.85 (2.62, 37.00) | 9.43 (2.32, 38.40) |
| Somatosensory dysfunction |  |  |  |  |
| 0 | ref | ref | ref | ref |
| Mild | 4.10 (2.74, 6.13) | 5.82 (4.11, 8.24) | 2.72 (2.06, 3.58) | 4.21 (2.93, 6.07) |
| Moderate | 42.76 (25.75, 71.01) | 35.79 (21.87, 58.58) | 16.43 (10.38, 26) | 31.34 (19.22, 51.08) |
| Severe | 118.17 (24.41, 572.07) | 94.60(19.64, 455.60) | 81.32 (10.23, 646.62) | 94.60 (19.64, 455.60) |
| Hypotension | 2.32 (0.78, 6.91) | 0.78 (0.18, 3.36) | 0.78 (0.23, 2.64) | 0.90 (0.21, 3.86) |
| Hypertension | 1.45 (1.05, 1.99) | 2.08 (1.55, 2.79) | 1.40 (1.10, 1.78) | 1.30 (0.96, 1.75) |
| Hyperlipidemia | 1.25 (0.80, 1.96) | 1.36 (0.92, 2.01) | 0.97 (0.67, 1.39) | 1.01 (0.64, 1.58) |
| Cardiovascular disease | 2.09 (1.43, 3.06) | 2.05 (1.45, 2.89) | 1.50 (1.09, 2.06) | 1.94 (1.35, 2.80) |
| Cerebrovascular disease | 3.32 (2.11, 5.21) | 3.66 (2.43, 5.52) | 2.45 (1.66, 3.63) | 2.66 (1.69, 4.19) |
| Linguistic incompetence | 10.85 (6.19, 19.03) | 8.55 (4.91, 14.89) | 6.56 (3.79, 11.36) | 9.22 (5.28, 16.11) |
| Varicose veins | 1.98 (0.92, 4.28) | 1.71 (0.82, 3.56) | 0.93 (0.43, 1.98) | 1.98 (0.95, 4.12) |
| Diabetes | 1.32 (0.85, 2.06) | 1.53 (1.05, 2.25) | 1.15 (0.81, 1.63) | 1.30 (0.85, 1.97) |
| Chronic bronchitis | 1.21 (0.59, 2.44) | 1.14 (0.60, 2.17) | 1.21 (0.70, 2.08) | 1.46 (0.78, 2.74) |
| Asthma | 0.91 (0.21, 3.87) | 1.07 (0.32, 3.61) | 1.58 (0.63, 3.96) | 2.28 (0.85, 6.12) |
| Arthritis | 1.20 (0.73, 1.96) | 1.28 (0.83, 1.98) | 1.40 (0.97, 2.01) | 1.43 (0.92, 2.22) |
| Osteoporosis | 1.67 (1.05, 2.65) | 1.28 (0.82, 2.00) | 1.35 (0.92, 1.96) | 1.34 (0.84, 2.13) |
| Intervertebral disc herniation | 1.03 (0.51, 2.08) | 1.19 (0.65, 2.16) | 0.87 (0.50, 1.53) | 1.00 (0.51, 1.96) |
| Hemorrhoids | 0.98 (0.50, 1.91) | 0.90 (0.49, 1.66) | 0.78 (0.46, 1.35) | 0.75 (0.37, 1.50) |
| Prostate hypertrophy | 1.40 (0.71, 2.76) | 2.02 (1.17, 3.50) | 1.06 (0.60, 1.87) | 1.66 (0.90, 3.05) |

Table S7 Univariate logistic regression analysis on individual items of dynamic balance (Continued)

| Variable | Ⅲ 13 | Ⅲ 14 | Ⅲ 15 | Ⅲ 16 |
| --- | --- | --- | --- | --- |
| Gender | 1.05 (0.79, 1.40) | 0.79 (0.54, 1.14) | 0.95 (0.67, 1.34) | 1.08 (0.80, 1.45) |
| Age (yrs) |  |  |  |  |
| 60- | ref | ref | ref | ref |
| 65- | 1.91 (1.01, 3.61) | 1.85 (0.82, 4.19) | 2.27 (1.07, 4.82) | 1.50 (0.77, 2.91) |
| 70- | 2.34 (1.22, 4.51) | 1.83 (0.76, 4.39) | 1.93 (0.85, 4.39) | 2.85 (1.51, 5.38) |
| 75- | 7.18 (3.97, 13.00) | 5.58 (2.59, 12.03) | 5.21 (2.50, 10.90) | 6.06 (3.32, 11.07) |
| 80- | 12.40 (6.69, 22.99) | 10.43 (4.78, 22.78) | 12.52 (6.02, 26.07) | 10.93 (5.86, 20.39) |
| 85-97 | 25.66 (13.47, 48.88) | 20.82 (9.46, 45.85) | 22.58 (10.64, 47.91) | 22.77 (11.92, 43.47) |
| Education |  |  |  |  |
| Elementary school or below | 3.88 (2.57, 5.86) | 3.59 (2.21, 5.84) | 3.78 (2.39, 5.96) | 3.11 (2.07, 4.66) |
| Middle or high school | 1.28 (0.91, 1.78) | 0.83 (0.53, 1.28) | 0.86 (0.58, 1.29) | 0.81 (0.58, 1.14) |
| College or above | ref | ref | ref | ref |
| Income | 0.77 (0.67, 0.89) | 0.90 (0.75, 1.08) | 0.92 (0.78, 1.09) | 0.87 (0.75, 1.00) |
| BMI (kg/m^2^) |  |  |  |  |
| Underweight (<18.5) | 2.27 (1.15, 4.49) | 2.36 (1.03, 5.41) | 2.94 (1.39, 6.22) | 3.02 (1.58, 5.77) |
| Overweight or obesity [24.0-) | 1.68 (1.25, 2.25) | 1.45 (0.99, 2.12) | 1.77 (1.24, 2.52) | 1.61 (1.19, 2.19) |
| Normal [18.5-24.0) | ref | ref | ref | ref |
| Exercise (times/week, >10 min/time) |  |  |  |  |
| 0- | ref | ref | ref | ref |
| 1- | 0.40 (0.28, 0.58) | 0.40 (0.25, 0.64) | 0.36 (0.23, 0.55) | 0.35 (0.24, 0.50) |
| 4- | 0.20 (0.11, 0.36) | 0.28 (0.15, 0.54) | 0.13 (0.06, 0.29) | 0.20 (0.11, 0.35) |
| 7- | 0.18 (0.12, 0.26) | 0.11 (0.06, 0.19) | 0.09 (0.05, 0.16) | 0.09 (0.06, 0.15) |
| Smoking | 1.25 (0.89, 1.75) | 1.66 (1.10, 2.50) | 1.66 (1.13, 2.43) | 1.35 (0.96, 1.90) |
| Vision (m) |  |  |  |  |
| 4- | ref | ref | ref | ref |
| 1- | 2.99 (2.17, 4.10) | 4.31 (2.82, 6.59) | 4.72 (3.16, 7.05) | 4.47 (3.18, 6.29) |
| 0- | 4.46 (2.91, 6.84) | 6.56 (3.84, 11.18) | 8.03 (4.90, 13.15) | 8.36 (5.44, 12.84) |
| Eye diseases | 1.47 (0.94, 2.30) | 1.31 (0.72, 2.38) | 1.39 (0.80, 2.39) | 1.44 (0.91, 2.30) |
| Hearing impairment |  |  |  |  |
| 0 | ref | ref | ref | ref |
| Mild | 4.46 (3.23, 6.15) | 8.16 (5.37, 12.40) | 6.17 (4.21, 9.04) | 6.63 (4.77, 9.22) |
| Moderate | 12.31 (6.96, 21.78) | 17.35 (9.03, 33.32) | 13.44 (7.20, 25.09) | 14.70 (8.22, 26.28) |
| Severe | 11.42 (3.02, 43.18) | 10.79 (2.17, 53.53) | 13.44 (3.28, 55.09) | 9.19 (2.26, 37.40) |
| Somatosensory dysfunction |  |  |  |  |
| 0 | ref | ref | ref | ref |
| Mild | 3.92 (2.77, 5.54) | 4.47 (2.75, 7.28) | 7.36 (4.58, 11.84) | 6.57 (4.50, 9.59) |
| Moderate | 35.21 (21.59, 57.41) | 39.08 (22.55, 67.70) | 61.67 (35.13, 108.27) | 52.44 (31.30, 87.86) |
| Severe |  | 110.71 (27.26, 449.69) | 79.56 (21.18, 298.80) | 120.89 (24.96, 585.56) |
| Hypotension | 1.26 (0.37, 4.27) | 0.71 (0.09, 5.31) | 1.26 (0.29, 5.42) | 1.93 (0.65, 5.73) |
| Hypertension | 1.52 (1.14, 2.03) | 1.59 (1.09, 2.31) | 1.69 (1.19, 2.39) | 1.50 (1.12, 2.02) |
| Hyperlipidemia | 1.12 (0.74, 1.70) | 1.17 (0.69, 2.00) | 1.10 (0.67, 1.82) | 1.29 (0.85, 1.94) |
| Cardiovascular disease | 1.90 (1.34, 2.70) | 2.31 (1.50, 3.55) | 2.10 (1.40, 3.17) | 2.25 (1.58, 3.20) |
| Cerebrovascular disease | 2.70 (1.75, 4.17) | 4.37 (2.70, 7.08) | 3.96 (2.49, 6.29) | 4.69 (3.13, 7.04) |
| Linguistic incompetence | 8.71 (5.00, 15.16) | 12.99 (7.27, 23.20) | 10.56 (5.95, 18.74) | 8.81 (5.05, 15.38) |
| Varicose veins | 1.99 (0.99, 4.02) | 1.66 (0.65, 4.25) | 2.06 (0.91, 4.64) | 2.46 (1.24, 4.85) |
| Diabetes | 1.39 (0.94, 2.06) | 1.27 (0.75, 2.13) | 1.26 (0.77, 2.04) | 1.24 (0.81, 1.88) |
| Chronic bronchitis | 2.00 (1.16, 3.46) | 1.33 (0.60, 2.93) | 1.10 (0.50, 2.43) | 2.19 (1.26, 3.79) |
| Asthma | 4.58 (2.01, 10.40) | 2.91 (0.99, 8.60) | 3.22 (1.19, 8.66) | 2.78 (1.10, 7.00) |
| Arthritis | 1.44 (0.94, 2.19) | 1.27 (0.73, 2.23) | 1.22 (0.72, 2.07) | 1.04 (0.64, 1.68) |
| Osteoporosis | 1.17 (0.74, 1.85) | 2.01 (1.21, 3.34) | 1.64 (1.00, 2.70) | 2.09 (1.39, 3.14) |
| Intervertebral disc herniation | 1.21 (0.66, 2.20) | 0.96 (0.41, 2.23) | 1.61 (0.84, 3.08) | 1.20 (0.64, 2.22) |
| Hemorrhoids | 0.42 (0.18, 0.97) | 0.66 (0.26, 1.65) | 1.06 (0.53, 2.14) | 1.00 (0.54, 1.85) |
| Prostate hypertrophy | 2.06 (1.19, 3.56) | 1.59 (0.75, 3.36) | 1.93 (1.00, 3.72) | 1.91 (1.07, 3.39) |
